# Significance of Plasmodium berghei Amino Acid Transporter 1 in Food Vacuole Functionality and Its Association with Cerebral Pathogenesis

**DOI:** 10.1128/spectrum.04943-22

**Published:** 2023-03-28

**Authors:** Aditya Anand, Manjunatha Chandana, Sourav Ghosh, Rahul Das, Nalini Singh, Pradeep Mini Vaishalli, Nagavara Prasad Gantasala, Govindarajan Padmanaban, Viswanathan Arun Nagaraj

**Affiliations:** a Infectious Disease Biology, Institute of Life Sciences, Bhubaneswar, Odisha, India; b Regional Centre for Biotechnology, Faridabad, Haryana, India; c School of Biotechnology, Kalinga Institute of Industrial Technology, Bhubaneswar, Odisha, India; d Advanced Technology Platform Centre, Regional Centre for Biotechnology, Faridabad, Haryana, India; e Department of Biochemistry, Indian Institute of Science, Bangalore, Karnataka, India; CSIR-Institute of Microbial Technology

**Keywords:** malaria, *Plasmodium berghei*, amino acid transporter 1, food vacuole, hemoglobin, hemozoin, experimental cerebral malaria

## Abstract

The food vacuole plays a central role in the blood stage of parasite development by digesting host hemoglobin acquired from red blood cells and detoxifying the host heme released during hemoglobin digestion into hemozoin. Blood-stage parasites undergo periodic schizont bursts, releasing food vacuoles containing hemozoin. Clinical studies in malaria-infected patients and *in vivo* animal studies have shown the association of hemozoin with disease pathogenesis and abnormal host immune responses in malaria. Here, we perform a detailed *in vivo* characterization of putative Plasmodium berghei amino acid transporter 1 localized in the food vacuole to understand its significance in the malaria parasite. We show that the targeted deletion of amino acid transporter 1 in Plasmodium berghei leads to a swollen food vacuole phenotype with the accumulation of host hemoglobin-derived peptides. Plasmodium berghei amino acid transporter 1-knockout parasites produce less hemozoin, and the hemozoin crystals display a thin morphology compared with wild-type parasites. The knockout parasites show reduced sensitivity to chloroquine and amodiaquine by showing recrudescence. More importantly, mice infected with the knockout parasites are protected from cerebral malaria and display reduced neuronal inflammation and cerebral complications. Genetic complementation of the knockout parasites restores the food vacuole morphology with hemozoin levels similar to that of wild-type parasites, causing cerebral malaria in the infected mice. The knockout parasites also show a significant delay in male gametocyte exflagellation. Our findings highlight the significance of amino acid transporter 1 in food vacuole functionality and its association with malaria pathogenesis and gametocyte development.

**IMPORTANCE** Food vacuoles of the malaria parasite are involved in the degradation of red blood cell hemoglobin. The amino acids derived from hemoglobin degradation support parasite growth, and the heme released is detoxified into hemozoin. Antimalarials such as quinolines target hemozoin formation in the food vacuole. Food vacuole transporters transport hemoglobin-derived amino acids and peptides from the food vacuole to the parasite cytosol. Such transporters are also associated with drug resistance. Here, we show that the deletion of amino acid transporter 1 in Plasmodium berghei leads to swollen food vacuoles with the accumulation of hemoglobin-derived peptides. The transporter-deleted parasites generate less hemozoin with thin crystal morphology and show reduced sensitivity to quinolines. Mice infected with transporter-deleted parasites are protected from cerebral malaria. There is also a delay in male gametocyte exflagellation, affecting transmission. Our findings uncover the functional significance of amino acid transporter 1 in the life cycle of the malaria parasite.

## INTRODUCTION

Malaria is a life-threatening disease that imposes a tremendous burden on global health. In 2021, 247 million cases and 619,000 deaths have been reported for malaria, and the most vulnerable populations represent children under 5 years of age and pregnant women (1). Among the five species causing human malaria, Plasmodium falciparum (*Pf*) is responsible for more than 90% of malaria mortality. Malaria-infected individuals experience mild to severe disease with a wide range of manifestations that include periodic fevers, anemia, respiratory distress syndrome, multiorgan failure, neurological complications, coma, and death. Cerebral malaria (CM) is the deadliest outcome with a high mortality rate, and about one-third of patients that survive cerebral malaria have a higher risk of neurological and cognitive deficits, behavioral difficulties, and epilepsy (2). Despite the intense global efforts to prevent malaria transmission and the existing WHO-recommended artemisinin-based combination therapies (ACTs) to treat malaria, malaria elimination and eradication remain a challenge. Since 2015, the global incidence of malaria has plateaued without any further significant reduction, raising concerns about rolling back malaria. In fact, there has been a considerable increase in malaria incidence, especially during the last 3 to 4 years. Various aspects, such as the emergence of drug-resistant parasites and insecticide-resistant *Anopheline* mosquitoes, a dearth of adjunct therapies for treating severe and cerebral malaria, and the implementation of an effective vaccination strategy with the recently WHO-approved RTS, S vaccine, should be a priority to control malaria (3). A detailed understanding of parasite biology and underlying mechanisms of parasite virulence and disease pathogenesis is also required to identify new targets for therapeutic interventions.

The food vacuole (FV) is an acidic lysosome-like organelle present in blood-stage parasites, where the endocytosed host hemoglobin (Hb) is digested by FV proteases (4). Besides feeding the parasites with Hb-derived amino acids, host Hb digestion provides space for the parasites to grow and maintains the osmotic stability of the infected red blood cells (RBCs) (5). The host heme released during Hb digestion is detoxified into hemozoin (Hz), and antimalarials, especially the quinolines, are known to inhibit Hz formation (6). FVs containing Hz are released into the bloodstream during periodic schizont bursts that occur at the end of each asexual replication cycle. Hz is a malarial pathogen-associated molecular pattern (PAMP) that plays an important role in disease pathogenesis (7). During the course of infection, Hz accumulation can be detected in various organs, including the spleen, liver, lungs, and brain (8). Phagocytotic cells, such as macrophages, dendritic cells, monocytes, and neutrophils, can readily engulf Hz and FVs containing Hz, and the levels of phagocytosed Hz correlate with disease severity (9). Hz and Hz-bound nucleic acids are recognized by Toll-like receptors, cyclic GMP-AMP synthase (cGAS), etc., leading to a severe proinflammatory response through NOD-like receptor protein 3 (NLRP3) inflammasome activation and interleukin-1β (IL-1β) production (10–12). Hz can also activate complement and intrinsic clotting pathways and lead to oxidative bursts and free radical production in innate immune cells (13, 14). Further, Hz can induce endothelial activation by upregulating the expression of cytoadherence molecules, such as CD36, ICAM1, etc., and promote aberrant host immune responses, parasite sequestration, and systemic and neuronal inflammation (15, 16).

Parasite FVs consist of a number of proteins that coordinate the molecular processes associated with Hb uptake and digestion and Hz formation (17). These include V-type H^+^ ATPases that maintain the acidic pH of FVs (18); histidine-rich protein-2, lipocalin-like protein PV5, and heme detoxification protein (HDP) that are required for Hz formation (19–21); aspartic and cysteine proteases such as plasmepsins and falcipains that facilitate the digestion of Hb into peptides (22, 23); metallopeptidases such as falcilysin (24); proteins involved in vesicular trafficking such as Rab GTPases (25); proteins trafficked to the parasite membrane, parasitophorous vacuolar membrane, and RBC cytosol that are endocytosed along with Hb; chaperones; metabolic enzymes such as glyceraldehyde-3-phosphate dehydrogenase (GAPDH), aldolase, etc.; and transporters (26). Because FVs are involved in Hb digestion, transporters present in this organelle assume significance in terms of transporting the peptides and free amino acids to the parasite cytosol. The peptides transported from FVs to the parasite cytosol are converted into free amino acids by the action of aminopeptidases (27, 28). Of the transporters present in FVs, chloroquine resistance transporter (CRT) and multidrug resistance protein 1 (MDR1) have been studied in detail because of their demonstrated association with antimalarial resistance (29, 30). Various studies have characterized genetic polymorphisms of CRT and MDR1 in the context of drug resistance, parasite fitness, and FV functionality (31, 32). It has also been shown that *Pf*CRT transports host-derived peptides of 4 to 11 residues (33). Here, we examine the *in vivo* functional significance of another putative FV transporter, amino acid transporter 1 (AAT1), in P. berghei (*Pb*) by performing targeted gene deletion. Our results provide new insights into the role of parasite AAT1 in maintaining optimal FV activity and Hz formation and its association with cerebral pathogenesis.

## RESULTS

### AAT1 is an FV-localized protein conserved across the *Plasmodium* species.

**FIG 1:**
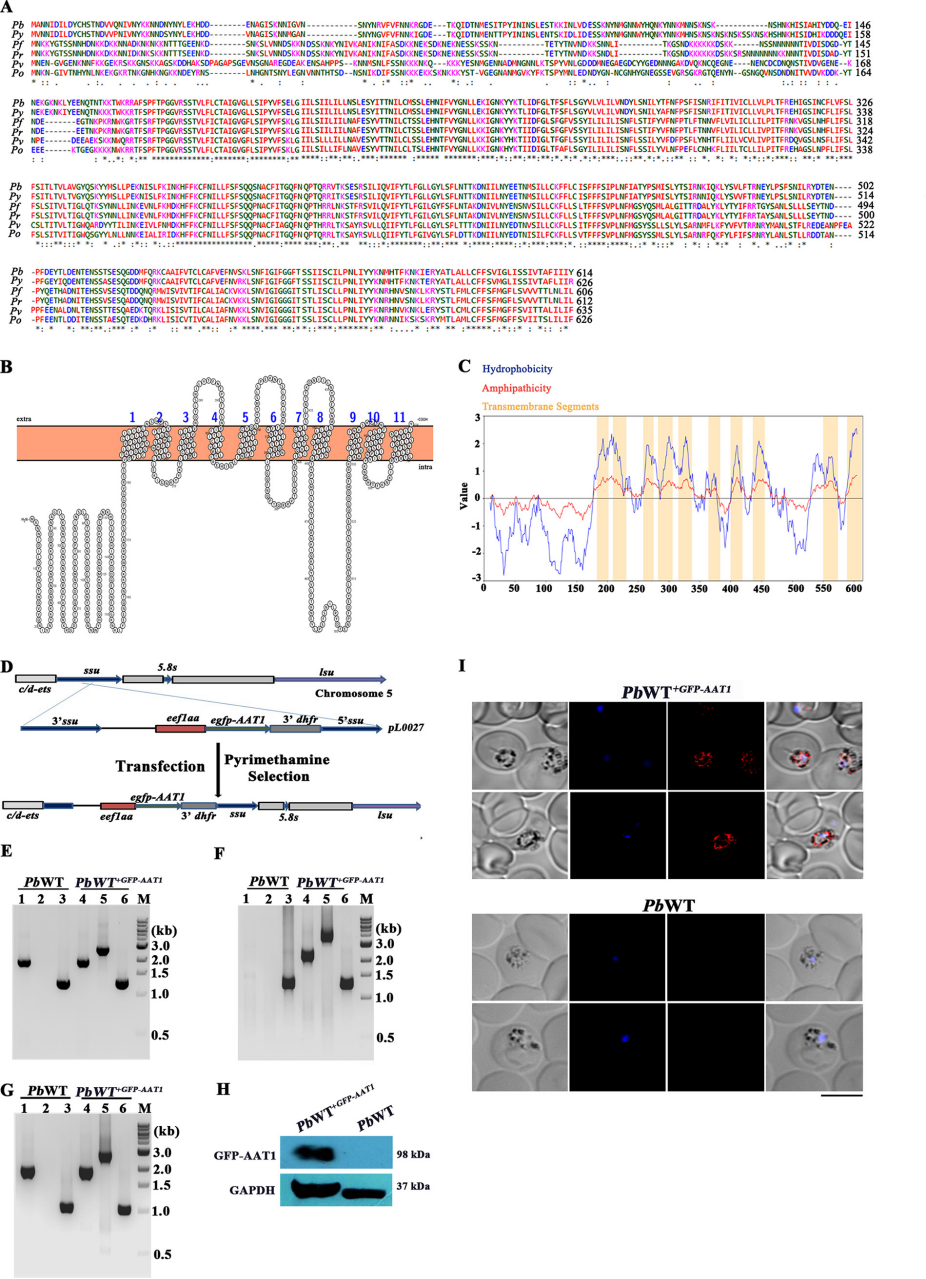
Bioinformatics analyses and localization of *Pb*AAT1. (A) Multiple sequence alignment of AAT1 from *Plasmodium* species. Amino acids conserved across the species are highlighted with asterisks (*). (B) Transmembrane topology of *Pb*AAT1 predicted by PROTTER; extra, extracellular membrane; intra, intracellular membrane. (C) Hydropathy (blue line) and amphipathicity (red line) index plots of *Pb*AAT1 using the WHAT tool (JNET program). Orange bars denote transmembrane segments predicted by the MEMSAT program. (D) Schematic representation of the single-crossover recombination strategy followed to generate *Pb*WT*^+GFP-AAT1^* parasites. (E) Genomic DNA PCR confirmation for the presence of *GFP-AAT1* in *Pb*WT*^+GFP-AAT1^* parasites; lanes 1 and 4, PCR amplification products of *AAT1* (1.93 kb) using *AAT1*-specific forward and reverse primers; lanes 2 and 5, PCR amplification products of *GFP-AAT1* (2.66 kb) using *GFP*-specific forward primer and *AAT1*-specific reverse primer; lanes 3 and 6, *GAPDH* control (1.25 kb). (F) Genomic DNA PCR confirmation for site-specific integration of *GFP-AAT1* in *Pb*WT*^+GFP-AAT1^* parasites; lanes 1 and 4, integration at the *c-ssurRNA* locus confirmed by amplification using L665 (*Pb*DHFR 3′ UTR-specific) and L740 (5.8 S-specific) primers (2.16 kb); lanes 2 and 5, integration at the *c-ssurRNA* locus confirmed by amplification using *Pb*AAT1 internal forward and L740 primers (3.38 kb); lanes 3 and 6, *GAPDH* control (1.25 kb); lane M, 1-kb ladder. (G) RT-PCR analysis of *GFP-AAT1* expression in *Pb*WT*^+GFP-AAT1^* parasites; lanes 1 and 4, RT-PCR amplification product of *AAT1* (1.84 kb) using *AAT1*-specific forward and reverse primers; lanes 2 and 5, RT-PCR amplification product of *GFP-AAT1* (2.57 kb) using *GFP*-specific forward primer and *AAT1*-specific reverse primer; lanes 3 and 6, *GAPDH* control (1.0 kb). (H) Western blotting of GFP-AAT1 protein expression in *Pb*WT*^+GFP-AAT1^* parasites; 150 μg of total protein was used. (I) Indirect immunofluorescence analysis of GFP-AAT1 localization in *Pb*WT*^+GFP-AAT1^* parasites. Images were captured using a 63× lens objective; scale bar = 5 μm.

AAT1 is conserved across the *Plasmodium* species and infects humans, primates, rodents, and birds. The P. berghei genome encodes a putative AAT1 (PBANKA_1128300) consisting of 614 amino acids. Its orthologue in P. falciparum, *Pf*AAT1 (PF3D7_0629500), has been shown to localize in the FV, and mutations in *Pf*AAT1 confer resistance against three diverse compounds from Medicines for Malaria Venture (MMV) (34); a genome-wide association analysis study has also suggested its association with chloroquine resistance (35). Multiple sequence alignment performed for AAT1 of human (P. falciparum, P. vivax [*Pv*], and *P. ovale* [*Po*]), primate (*P. reichenowi* [Pr]), and rodent (P. berghei and P. yoelii [Py]) parasites showed ~51% identity and ~67% similarity across the species (Fig. 1A). An interactive protein feature visualization using the PROTTER and web-based hydropathy, amphipathicity, and topology (WHAT) tools predicted 10 to 11 transmembrane domains for *Pb*AAT1 (Fig. 1B and C). Our attempts to express recombinant *Pb*AAT1 for raising antibodies and to tag the endogenous *Pb*AAT1 with reporter genes turned out to be unsuccessful. Therefore, to examine the localization of AAT1 in P. berghei, we introduced a copy of AAT1 tagged at the N terminus with green fluorescence protein (GFP) into the small subunit rRNA (*ssurRNA*) locus of P. berghei wild-type (*Pb*WT) parasites (Fig. 1D). For this, luciferase present in the GFP-luciferase coding sequence of *pL0027* plasmid was replaced with AAT1, and the recombinant plasmid was transfected into *Pb*WT schizonts. The presence of *GFP-AAT1* in the transgenic parasites (*Pb*WT*^+GFP-AAT1^*), its site-specific integration in the *ssurRNA* locus, and the expression of *GFP-AAT1* RNA were confirmed by genomic DNA (Fig. 1E and F) and reverse transcription-PCR (RT-PCR) (Fig. 1G) analyses. Western blotting performed using GFP antibodies showed the expression of GFP-AAT1 in *Pb*WT*^+GFP-AAT1^* parasite lysates (Fig. 1H). Live fluorescence imaging performed with *Pb*WT*^+GFP-AAT1^* parasites showed a faint signal probably due to the loss of GFP fluorescence in the acidic environment of FVs (Fig. S1 in the supplemental material). Hence, we performed indirect immunofluorescence using GFP antibodies to examine its localization. The results obtained showed the colocalization of GFP-AAT1 with discrete FVs containing Hz in *Pb*WT*^+GFP-AAT1^* parasites (Fig. 1I), suggesting the FV localization of *Pb*AAT1.

### AAT1 is nonessential for *in vivo* growth of P. berghei asexual stages.

**FIG 2:**
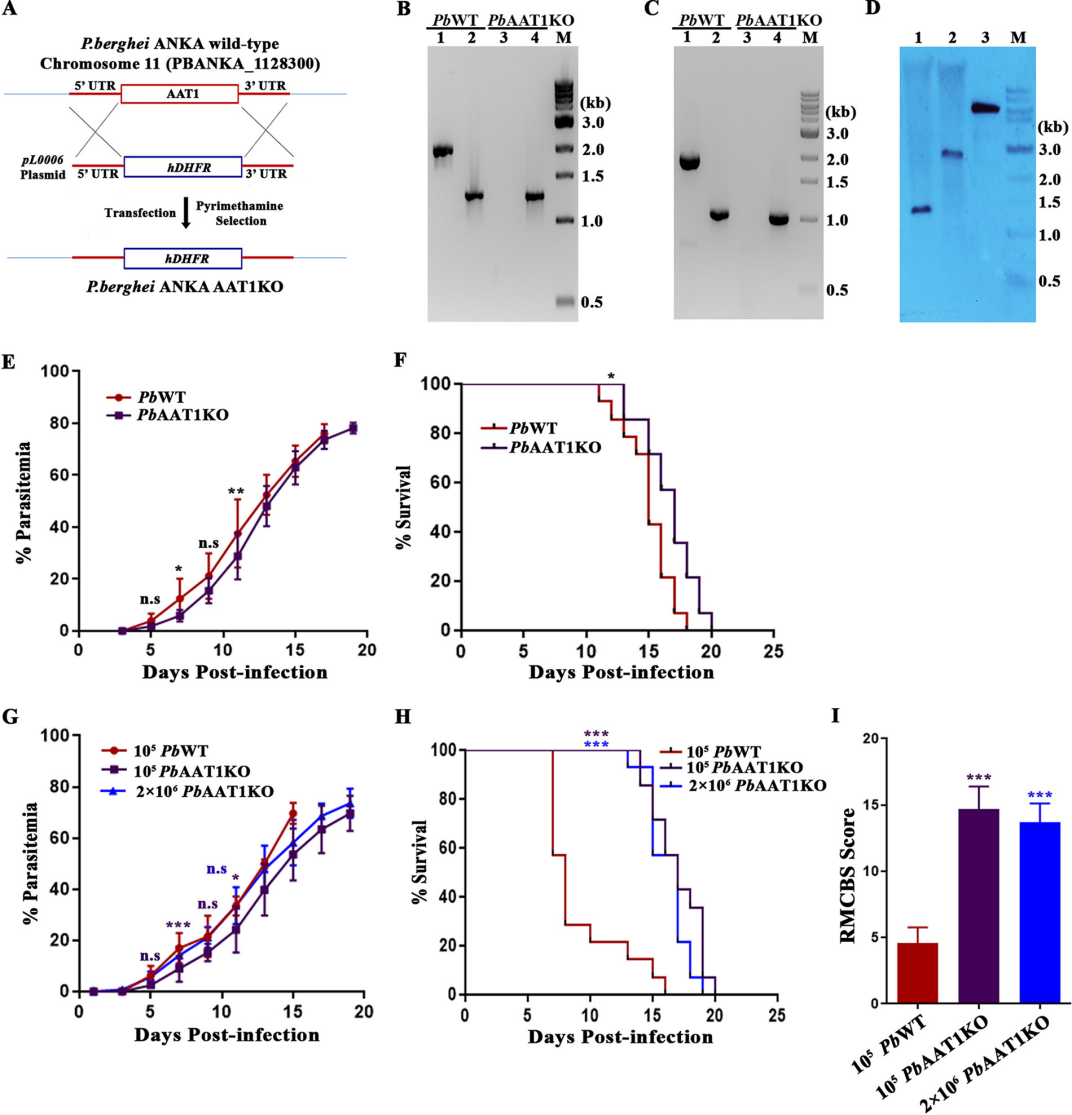
Generation of *Pb*AAT1KO parasites and their asexual-stage phenotype. (A) The double crossover recombination strategy followed to generate *Pb*AAT1KO parasites. (B) Genomic DNA PCR confirmation of *AAT1* deletion in *Pb*AAT1KO parasites; lanes 1 and 3, amplification of *AAT1* (1.93 kb); lanes 2 and 4, *GAPDH* control (1.25 kb). (C) RT-PCR confirmation of *AAT1* deletion in *Pb*AAT1KO parasites; lanes 1 and 3, amplification of *AAT1* (1.84 kb); lanes 2 and 4, *GAPDH* control (1.0 kb). (D) Southern blotting confirmation of site-specific integration in *Pb*AAT1KO parasites; lanes 1 and 2, genomic DNA isolated from *Pb*WT and *Pb*AAT1KO parasites, respectively; lane 3, recombinant plasmid used for transfection as a control to rule out the presence of episomes. The genomic DNA (5 μg) and plasmid (0.1 μg) samples were digested with SpeI and XhoI followed by hybridization with 3′-UTR-specific probe. *Pb*WT and *Pb*AAT1KO genomic DNA showed the corresponding hybridized fragments of sizes 1.2 kb and 2.5 kb, respectively. For recombinant plasmid, the fragment size was 5.8 kb. (E) Growth analysis of *Pb*WT (*n* = 14) and *Pb*AAT1KO (*n* = 14) parasites in BALB/c mice; 10^5^ parasites were used to initiate *Pb*WT and *Pb*AAT1KO parasite infections. The data (mean ± standard deviation [SD]) represent three different batches; n.s, not significant; *, *P < *0.05; **, *P < *0.01; ***, *P < *0.001. Data were analyzed by two-way ANOVA. (F) Survival curves of BALB/c mice infected with *Pb*WT and *Pb*AAT1KO parasites; *, *P* < 0.05. Data were analyzed by log-rank (Mantel-Cox) test. The data represent the mice used for growth analysis. (G) Growth analysis of *Pb*WT (*n* = 14) and *Pb*AAT1KO (*n* = 14) parasites in C57BL/6 mice; 10^5^ parasites were used to initiate *Pb*WT infections, and 10^5^ and 2 × 10^6^ parasites were used to initiate *Pb*AAT1KO infections. The data (mean ± SD) represent three different batches; n.s, not significant; *, *P < *0.05, **, *P < *0.01. Data were analyzed by two-way ANOVA. (H) Survival curves of C57BL/6 mice infected with *Pb*WT and *Pb*AAT1KO parasites; ***, *P* < 0.001. Data were analyzed by log-rank (Mantel-Cox) test. The data represent the mice used for growth analysis. (I) RMCBS score for mice infected with *Pb*WT (*n* = 10) and *Pb*AAT1KO (*n* = 10) parasites (mean ± SD); ***, *P < *0.001. Data were analyzed by two-way ANOVA.

Our next interest was to understand the significance of AAT1 in the asexual stages of P. berghei. For this, we performed targeted gene deletion for *Pb*AAT1 by transfecting *Pb*WT schizonts with *pL0006* plasmid with 5′ and 3′ untranslated regions (UTRs) of the *Pb*AAT1 gene flanking either side of a human dihydrofolate reductase (DHFR) selection cassette (Fig. 2A). The deletion of *AAT1* in *Pb*AAT1-knockout (*Pb*AAT1KO) parasites was confirmed by genomic DNA and RT-PCR analyses (Fig. 2B and C) and by Southern blotting (Fig. 2D). Growth curve analysis performed for the blood-stage infections in BALB/c mice through intraperitoneal injection of 10^5^ parasites suggested that the growth of *Pb*AAT1KO parasites was delayed by ~2 days compared with *Pb*WT parasites (Fig. 2E). There was also a significant delay of ~2 to 3 days in mortality due to anemia (Fig. 2F). A similar growth delay was also observed for *Pb*AAT1KO parasites in C57BL/6 mice (Fig. 2G), a mouse model for experimental cerebral malaria (ECM). Importantly, C57BL/6 mice infected with *Pb*AAT1KO parasites were protected from ECM. While 60 to 70% of *Pb*WT-infected mice died of ECM within day 10 postinfection, mice infected with *Pb*AAT1KO parasites survived beyond day 15 and died of anemia (Fig. 2H). ECM protection was also verified by injecting a higher number (2 × 10^6^) of *Pb*AAT1KO parasites to match their growth rate with *Pb*WT (Fig. 2G and H). The data suggested that ECM protection was not due to the delay in *Pb*AAT1KO parasite growth. The absence of cerebral pathogenesis was reflected in the higher rapid murine coma and behavior scale (RMCBS) score of *Pb*AAT1KO-infected mice (Fig. 2I). All these results indicated that *Pb*AAT1KO parasites were less virulent than *Pb*WT, and they did not induce cerebral pathogenesis.

### *Pb*AAT1KO asexual-stage parasites have swollen FVs with less hemozoin.

**FIG 3:**
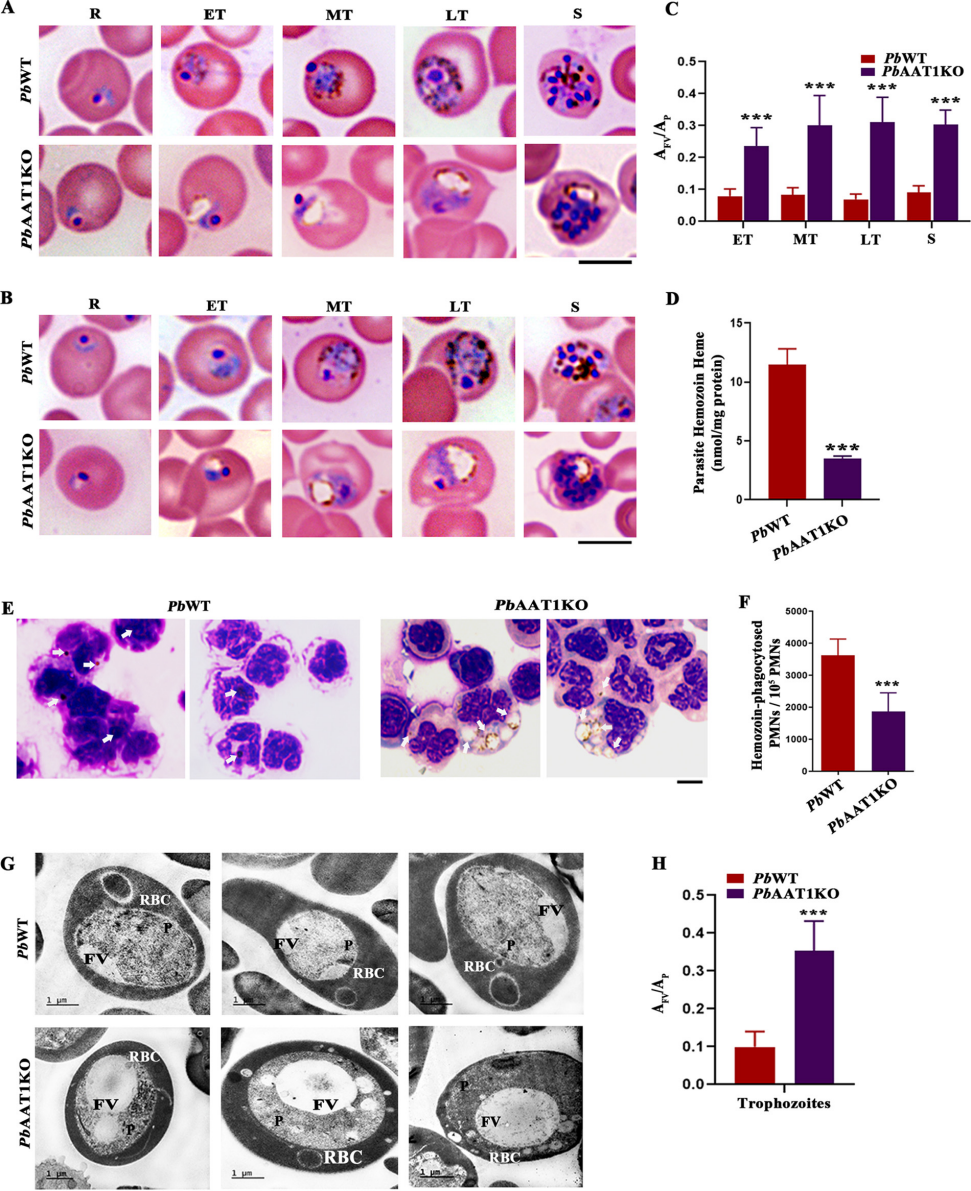
Characterization of *Pb*AAT1KO FVs. (A) Giemsa-stained images of *Pb*WT and *Pb*AAT1KO parasite-infected RBCs from BALB/c mice. (B) Giemsa-stained images of *Pb*WT and *Pb*AAT1KO parasite-infected RBCs from C57BL/6 mice. Brightfield images were captured using a 100× lens objective; scale bar = 5 μm. (C) The *A*_FV_/*A*_P_ values of *Pb*WT and *Pb*AAT1KO parasites measured from Giemsa-stained images. The data (mean ± SD) represent at least 25 independent measurements for the respective stages; ***, *P < *0.001. Data were analyzed by two-way ANOVA; R, rings; ET, early trophozoites; MT, midtrophozoites; LT, late trophozoites; S, schizonts. (D) Quantification of Hz levels in *Pb*WT (*n* = 6) and *Pb*AAT1KO parasites (*n* = 6). The data represent mean ± SD; ***, *P < *0.001. Data were analyzed by unpaired *t* test (two tailed). (E) Giemsa images of PMNs isolated from *Pb*WT- and *Pb*AAT1KO-infected mice showing engulfed FVs and Hz. Images were captured using a 100× lens objective; scale bar = 5 μm. (F) Number of Hz-containing polymorphonuclear leukocytes. The data (*n* = 6) represent mean ± SD; ***, *P < *0.001. Data were analyzed by unpaired *t* test (two tailed). (G) TEM images of *Pb*WT and *Pb*AAT1KO parasite-infected RBCs; scale bar = 1 μm; RBC, red blood cell; P, parasite; FV, food vacuole. (H) The *A*_FV_/*A*_P_ values of *Pb*WT and *Pb*AAT1KO trophozoites measured from TEM images. The data (mean ± SD) represent 15 independent measurements; ***, *P < *0.001. Data were analyzed by two-tailed paired *t* test.

Giemsa-stained images of *Pb*AAT1KO asexual-stage parasites from BALB/c and C57BL/6 mice showed swollen FVs (Fig. 3A and B). Starting from the early trophozoites, swollen FVs could be detected in all the asexual stages, although they were more prominent in mature trophozoites and schizonts with Hz crystals mainly distributed to the periphery of FVs. Similar observations were made in live and paraformaldehyde-fixed *Pb*AAT1KO parasites (Fig. S2A and B). The swollen FV phenotype of *Pb*AAT1KO parasites was consistent during repeated blood-stage passages and cryopreservations. For further characterization of swollen FVs, we performed *Pb*AAT1KO infections in C57BL/6 mice. The ratio of FV area with respect to the parasite area (*A*_FV_/*A*_P_) was ~2 to 3 times higher for *Pb*AAT1KO parasites (Fig. 3C). Biochemical quantification of Hz levels normalized with respect to the total protein showed an ~70% decrease in *Pb*AAT1KO parasites compared with *Pb*WT parasites (Fig. 3D). There was also a significant decrease in the Hz levels accumulated in organs such as the spleen, liver, lungs, and brain of *Pb*AAT1KO-infected mice (Fig. S2C to F). In agreement with ECM protection, decreases in brain Hz levels were ~2.5-times higher than the decreases observed in the spleen, liver, and lungs of *Pb*AAT1KO-infected mice. There was also a significant decrease in the number of Hz-containing polymorphonuclear leukocytes (PMNs) in mice infected with *Pb*AAT1KO parasites (Fig. 3E and F). Transmission electron microscopy (TEM) analyses suggested that the swollen *Pb*AAT1KO FVs were electron lucent, indicating the accumulation of solutes or smaller peptides (Fig. 3G). As observed in Giemsa-stained images, *A*_FV_/*A*_P_ measured for trophozoite stages in TEM analyses showed an ~3-fold increase for *Pb*AAT1KO parasites (Fig. 3H).

**FIG 4:**
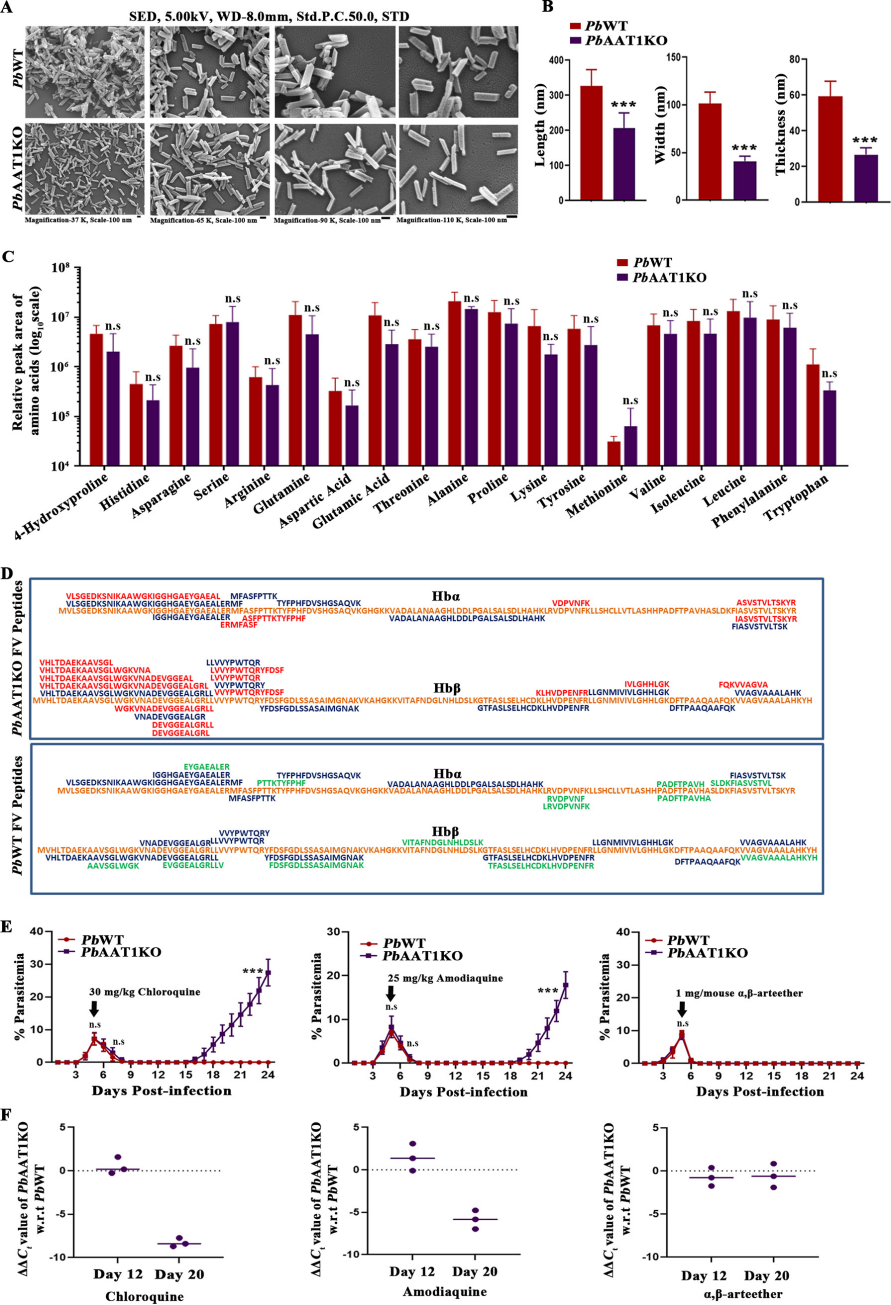
Hz crystal dimensions, peptide accumulation, and quinoline sensitivity in *Pb*AAT1KO parasites. (A) Scanning electron microscopy images of Hz crystals isolated from *Pb*WT and *Pb*AAT1KO parasites. The images were captured at four different magnifications; 37,000×, 65,000×, 90,000×, and 110,000× are shown; SED, secondary electron detector; WD, working distance; Std.P.C., standard probe current; STD, standard mode. (B) Dimensions of Hz crystals isolated from *Pb*WT and *Pb*AAT1KO parasites. The length, width, and thickness of Hz crystals were measured from scanning electron microscopy images. The data (mean ± SD) represent the values obtained from 100 independent crystals; ***, *P < *0.001. Data were analyzed by two-tailed unpaired *t* test. (C) The relative peak areas of the amino acids present in *Pb*WT and *Pb*AAT1KO FVs analyzed by LC-MS/MS. The data (mean ± SD) represent three independent preparations; n.s, not significant. Data were analyzed by two-way ANOVA. For each preparation, three FV pellets of *Pb*WT and *Pb*AAT1KO were pooled separately. The relative peak areas of the respective amino acids were normalized against a nonprotein amino acid citrulline. (D) Schematic representation of the peptides identified in *Pb*WT and *Pb*AAT1KO FVs by LC-MS/MS analysis. Four different FVs were pooled separately for *Pb*WT and *Pb*AAT1KO parasites. The α and β chains of mouse hemoglobin are represented in orange. Peptides unique for *Pb*AAT1KO FVs are highlighted in red. Peptides unique for *Pb*WT FVs are highlighted in green. Peptides commonly identified in *Pb*WT and *Pb*AAT1KO FVs are highlighted in blue. (E) Effect of quinolines (chloroquine and amodiaquine) and α,β-arteether on *Pb*AAT1KO-infected mice. Mice were inoculated with 10^5^ *Pb*WT and 2 × 10^6^ *Pb*AAT1KO parasites. The data (mean ± SD) represent two different batches of three mice each; n.s, not significant; ***, *P < *0.001. Data were analyzed by two-way ANOVA. (F) qPCR analysis of parasite load in *Pb*WT- and *Pb*AAT1KO-infected mice treated with chloroquine, amodiaquine, and α,β-arteether. The data represent three different mice each for *Pb*WT and *Pb*AAT1KO. The ΔΔ*C_t_* values obtained for parasite *GAPDH* with respect to mouse *GAPDH* are shown. w.r.t, with respect to.

Scanning electron microscopy analyses of Hz purified from *Pb*WT and *Pb*AAT1KO parasites showed that the Hz crystals of *Pb*AAT1KO parasites were morphologically thin compared with *Pb*WT Hz crystals (Fig. 4A). The measurement of Hz crystal dimensions isolated from *Pb*AAT1KO parasites suggested a significant reduction in the length, width, and thickness. In particular, there was an almost greater than 50% reduction in the width and thickness of *Pb*AAT1KO Hz crystals (Fig. 4B). To examine whether the swollen FV phenotype of *Pb*AAT1KO was due to the accumulation of any amino acid, we performed liquid chromatography-tandem mass spectrometry (LC-MS/MS) and high-performance liquid chromatography (HPLC) analyses for *Pb*WT and *Pb*AAT1KO FVs. The purified *Pb*AAT1KO FVs were pale brown in color compared with the dark brown color of *Pb*WT FVs, suggesting less Hz content in *Pb*AAT1KO parasites (Fig. S2G). The results obtained from LC-MS/MS and HPLC analyses suggested that there was no significant accumulation of amino acids in *Pb*AAT1KO FVs (Fig. 4C; Fig. S3A). We next examined the accumulation of peptides in *Pb*AAT1KO FVs, and for this, four independent FV preparations of *Pb*WT and *Pb*AAT1KO parasites were pooled separately. LC-MS/MS analysis of the peptides extracted from the FVs of *Pb*AAT1KO parasites suggested the accumulation of 19 unique peptides corresponding to Hb α and β chains that could not be detected in *Pb*WT FVs. The length of these accumulated peptides varied between 7 and 31 amino acid residues. Further, 13 peptides detected in *Pb*WT FVs were found to be absent in *Pb*AAT1KO FVs, and 15 peptides were common between *Pb*WT and *Pb*AAT1KO FVs (Fig. 4D; Data Set S1). The normalized total spectral intensity of all peptides corresponding to Hb α and β chains in *Pb*AAT1KO FV preparations was ~3 times higher than in WT FVs (Fig. S3B). However, the total spectral intensity of the common peptides was comparable between *Pb*WT and *Pb*AAT1KO FVs (Fig. S3B; Data Set S1), despite the individual variations observed in the fold changes (Fig. S3C). Importantly, the total spectral intensity of the unique *Pb*AAT1KO FV peptides accounted for ~75% of the entire peptides. In contrast, the total spectral intensity of the unique *Pb*WT FV peptides accounted only for ~15% of all peptides (Fig. S3B). Similar results were also obtained with the MS2 spectral counts (Fig. S3B and C; Data Set S1). This in turn suggested that the swollen FV phenotype was due to the accumulation of the unique peptides identified in *Pb*AAT1KO FVs. All these findings suggested the accumulation of Hb-derived peptides and an impairment of Hb digestion in *Pb*AAT1KO FVs.

### Sensitivity of *Pb*AAT1KO parasites to quinolines.

Quinolines are known to interfere with Hz formation in the FVs (36). Since FV morphology and Hz formation were compromised in *Pb*AAT1KO parasites, it was of interest to examine the sensitivity of *Pb*AAT1KO parasites toward the quinolines chloroquine and amodiaquine. The treatment of *Pb*WT- and *Pb*AAT1KO-infected BALB/c mice with both drugs was initiated on day 5 postinfection, when blood parasitemia was ~7%, and continued until day 10 postinfection. The assessment of blood parasitemia by Giemsa-stained smears showed an initial parasite clearance that was comparable between *Pb*WT and *Pb*AAT1KO infections for chloroquine and amodiaquine. However, there was parasite recrudescence in *Pb*AAT1KO-infected mice but not in *Pb*WT-infected mice (Fig. 4E). The recrudescent parasites were detectable around days 15 to 18 postinfection, and *Pb*AAT1KO-infected mice treated with chloroquine and amodiaquine eventually died of anemia around days 30 to 35 postinfection. For a control, α,β-arteether treatment was performed, and, as expected, there was no difference between *Pb*WT- and *Pb*AAT1KO-infected mice. These results were also verified by quantitative PCR (qPCR) analysis performed on days 12 and 20 postinfection. While the difference in cycling threshold (ΔΔ*C_t_*) values of chloroquine- and amodiaquine-treated *Pb*AAT1KO-infected mice with respect to *Pb*WT-infected mice on day 12 were close to 0, the values obtained on day 20 were around −5 to −8, suggesting a higher parasite load of more than 32 times due to recrudescence. In the case of α,β-arteether treatment, ΔΔ*C_t_* values on day 20 were also close to 0 because there was no recrudescence (Fig. 4F). These results suggest the lower sensitivity of *Pb*AAT1KO parasites to quinolines.

### Reduced cerebral pathogenesis in *Pb*AAT1KO-infected mice.

**FIG 5:**
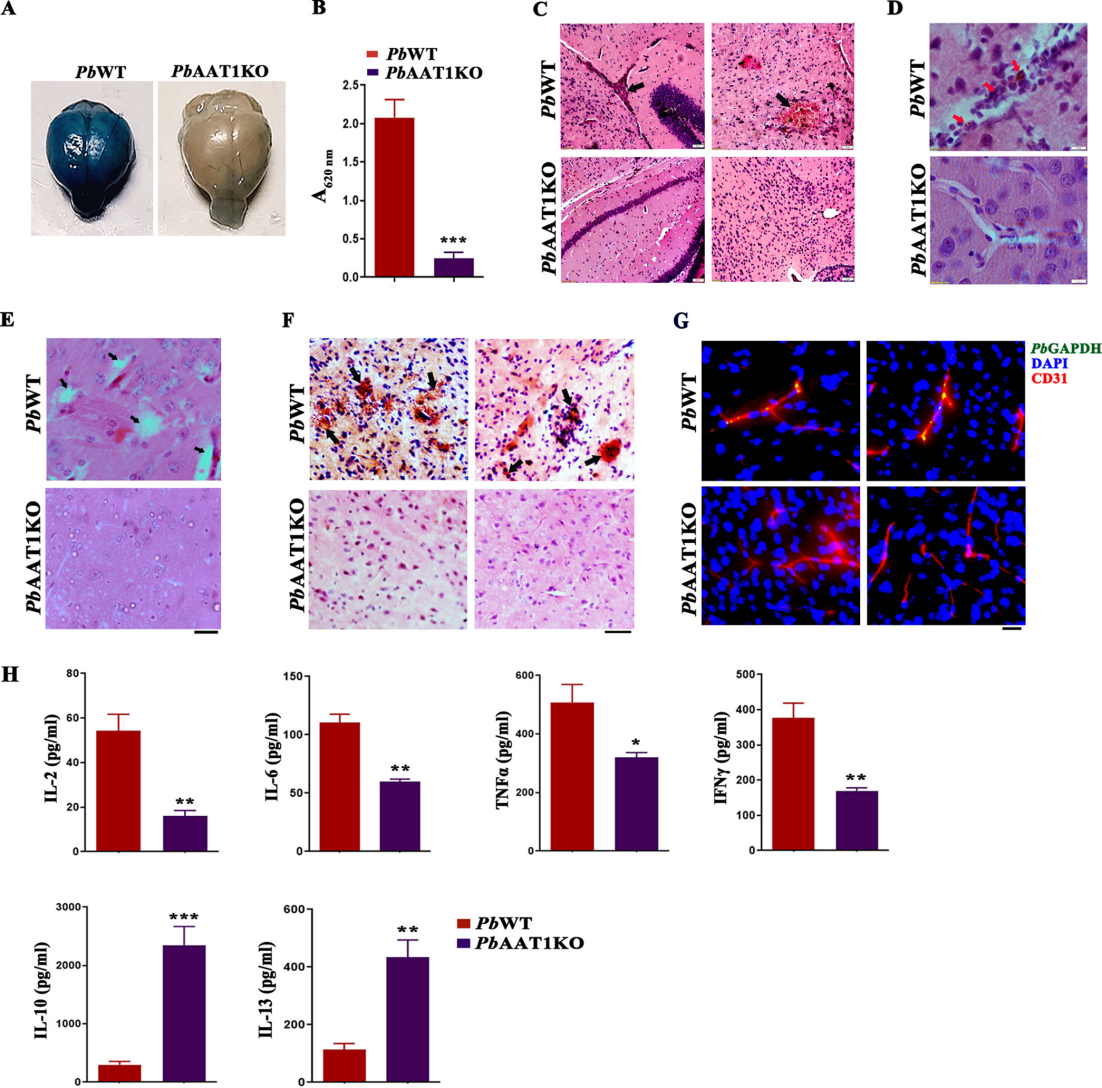
Cerebral pathogenesis in *Pb*AAT1KO-infected C57BL/6 mice. (A) Evans blue extravasation in the brain samples of *Pb*WT- and *Pb*AAT1KO-infected mice on day 8 postinfection. (B) Quantification of Evans blue extravasation. The data (mean ± SD) represent six different mice; ***, *P < *0.001. Data were analyzed by two-tailed unpaired *t* test; *A*_620 nm_, absorbance at 620 nm. (C) H&E staining of brain sections from *Pb*WT- and *Pb*AAT1KO-infected mice. Black arrows represent thrombosed blood vessels, intracerebral hemorrhages, and vascular leakage. Images were captured using a 10× lens objective; scale bar = 50 μm. (D) H&E-stained blood vessels of *Pb*WT- and *Pb*AAT1KO-infected mice. Red arrows represent leukocyte accumulation with phagocytosed Hz in the occluded vessels. Images were captured using a 60× lens objective; scale bar = 10 μm. (E) H&E-stained perivascular brain sections of *Pb*WT- and *Pb*AAT1KO-infected mice. Black arrows represent cerebral edema. Images were captured using a 40× lens objective; scale bar = 20 μm. (F) IgG immunoreactivity of *Pb*WT and *Pb*AAT1KO brain sections. Black arrows represent areas showing IgG immunoreactivity. Images were captured using a 10× lens objective; scale bar = 50 μm. (G) Accumulation of parasitized RBCs in the CD31^+^ brain vasculature of *Pb*WT- and *Pb*AAT1KO-infected mice. Images were captured using a 20× lens objective; scale bar = 20 μm. (H) Plasma cytokine levels of *Pb*WT- and *Pb*AAT1KO-infected mice. The data (mean ± standard error of the mean [SEM]) represent three different mice; *, *P < *0.05; **, *P < *0.01; ***, *P < *0.001. Data were analyzed by two-tailed unpaired *t* test.

In agreement with ECM protection, there was very little Evans blue extravasation in *Pb*AAT1KO-infected brain samples. This in turn suggested that the blood-brain barrier (BBB) integrity of *Pb*AAT1KO-infected mice was intact compared with *Pb*WT-infected mice (Fig. 5A and B). Hematoxylin and eosin (H&E) staining of brain sections prepared from *Pb*WT-infected mice showed thrombosed vessels, intracerebral hemorrhages, vascular leakage, leukocyte accumulation in the occluded vessels, and cerebral edema (Fig. 5C to E). Immunohistochemistry analyses of the brain sections from *Pb*WT-infected mice showed IgG immunoreactivity along with the detection of parasites in CD31^+^ vasculature (Fig. 5F and G). These characteristic features of cerebral pathogenesis could not be detected in the brain sections of *Pb*AAT1KO-infected mice (Fig. 5C to G). Plasma samples of *Pb*AAT1KO-infected mice showed a significant reduction in the levels of proinflammatory cytokines IL-2, IL-6, tumor necrosis factor-α (TNF-α), and interferon-γ (IFN-γ) and a significant increase in the levels of anti-inflammatory cytokines IL-10 and IL-13 (Fig. 5H).

**FIG 6:**
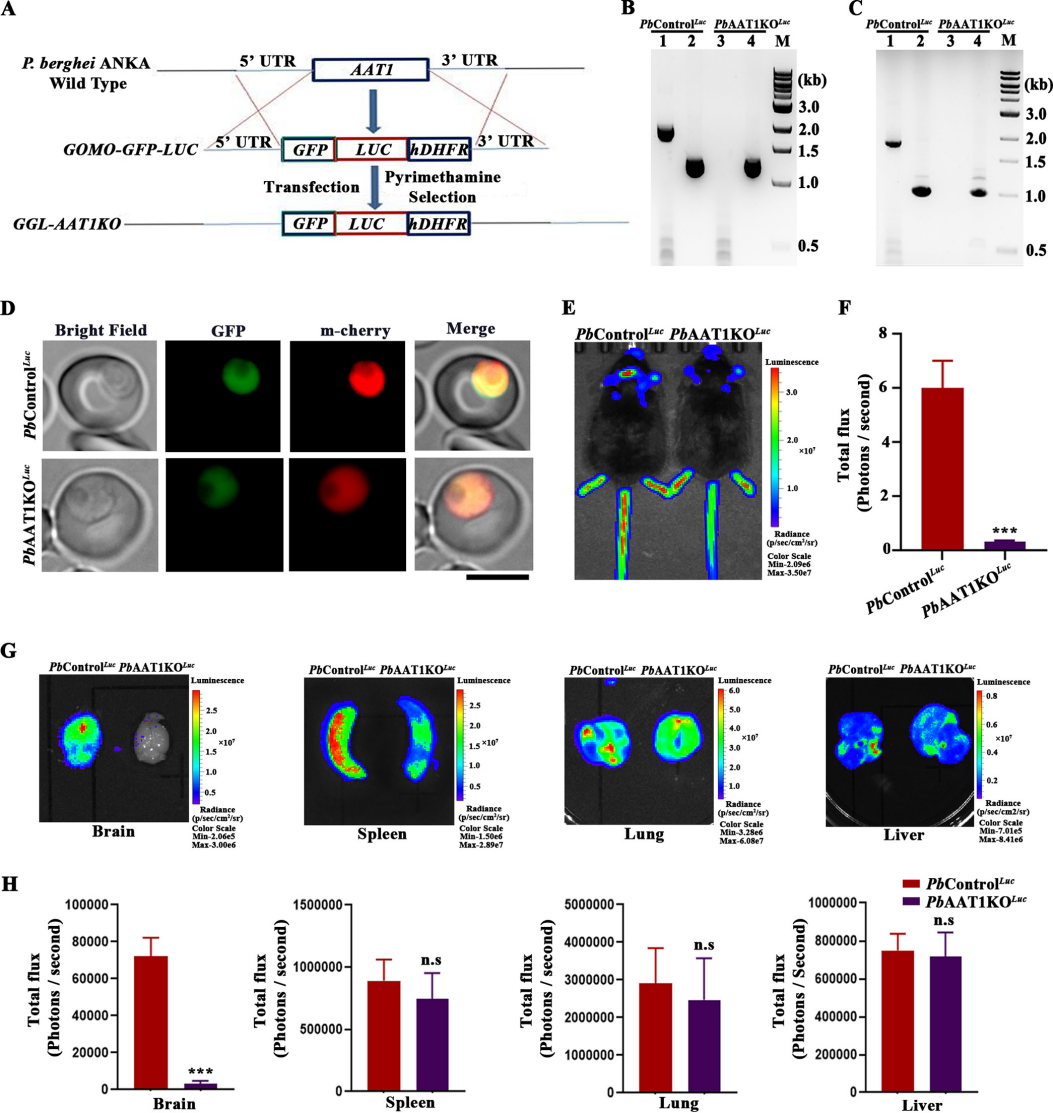
Bioluminescence imaging of *Pb*AAT1KO^Luc^-infected mice. (A) Double crossover recombination strategy followed to generate *Pb*AAT1KO^Luc^ parasites. (B) Genomic DNA PCR confirmation of *AAT1* deletion in *Pb*AAT1KO^Luc^ parasites; lanes 1 and 3, amplification of *AAT1* (1.93 kb); lanes 2 and 4, *GAPDH* control (1.25 kb). (C) RT-PCR confirmation of *AAT1* deletion in *Pb*AAT1KO^Luc^ parasites; lanes 1 and 3, amplification of *AAT1* (1.84 kb); lanes 2 and 4, *GAPDH* control (1.0 kb). (D) GFP and mCherry fluorescence of *Pb*Control^Luc^ and *Pb*AAT1KO^Luc^ parasites. Images were captured using a 100× lens objective; scale bar = 5 μm. (E) *In vivo* bioluminescence imaging of C57BL/6 mice infected with *Pb*Control^Luc^ and *Pb*AAT1KO^Luc^ parasites. (F) *In vivo* bioluminescence intensity of the brains of C57BL/6 mice infected with *Pb*Control^Luc^ and *Pb*AAT1KO^Luc^ parasites. The brain portions from whole-body imaging were selected as regions of interest. The data (mean ± SD) represent six different mice; ***, *P < *0.001. Data were analyzed by two-tailed unpaired *t* test. (G) *Ex vivo* bioluminescence of brain, spleen, lung, and liver isolated from *Pb*Control^Luc^- and *Pb*AAT1KO^Luc^-infected mice. (H) *Ex vivo* bioluminescence intensities of the isolated organs. The data (mean ± SD) represent three different mice; ***, *P < *0.001; n.s, not significant. Data were analyzed by two-tailed unpaired *t* test.

The reduced cerebral pathogenesis in *Pb*AAT1KO parasite-infected mice was also verified by *in vivo* and *ex vivo* bioluminescence studies. For this, *Pb*AAT1KO parasites expressing GFP-luciferase along with mCherry (*Pb*AAT1KO^Luc^) were generated by transfecting *Pb*WT schizonts with Gene Out Marker Out (GOMO)-GFP-luciferase plasmid containing 5′ and 3′ UTRs of the *Pb*AAT1 gene flanking either side of the human DHFR fused with yeast cytosine deaminase-uridyl phosphoribosyl transferase (hDHFR-yFCU) selection cassette (Fig. 6A). The targeted deletion of *AAT1* was confirmed by genomic DNA and RT-PCR analyses (Fig. 6B and C). *Pb*WT parasites transfected with GOMO-GFP-luciferase plasmid to replace the endogenous *ssurRNA* with *GFP-luciferase* were used as controls (*Pb*Control^Luc^). Live imaging studies suggested the presence of GFP and mCherry fluorescence in both *Pb*AAT1KO^Luc^ and *Pb*Control^Luc^ parasites (Fig. 6D). *Pb*AAT1KO^Luc^ parasites showed a similar delay in parasite growth and mortality in C57BL/6 mice as observed for *Pb*AAT1KO parasites (Fig. S4A and B). There was also ECM protection with a high RMCBS score (Fig. S4C). In agreement with this, *in vivo* bioluminescence signal was hardly detectable in the brains of mice infected with *Pb*AAT1KO^Luc^ parasites (Fig. 6E and F). *Ex vivo* bioluminescence analysis of brain samples isolated from infected mice showed a prominent signal for *Pb*Control^Luc^ parasites but not for *Pb*AAT1KO*^Luc^* parasites. However, there were no significant differences in the *ex vivo* bioluminescence signal of spleens, lungs, and livers between *Pb*Control^Luc^- and *Pb*AAT1KO^Luc^-infected mice (Fig. 6G and H). The results obtained with *Pb*AAT1KO^Luc^ parasites further confirmed the ECM protection and reduced cerebral pathogenesis in P. berghei parasites lacking AAT1.

### Inflammation analysis and immune profiling of brains in *Pb*AAT1KO-infected mice.

**FIG 7:**
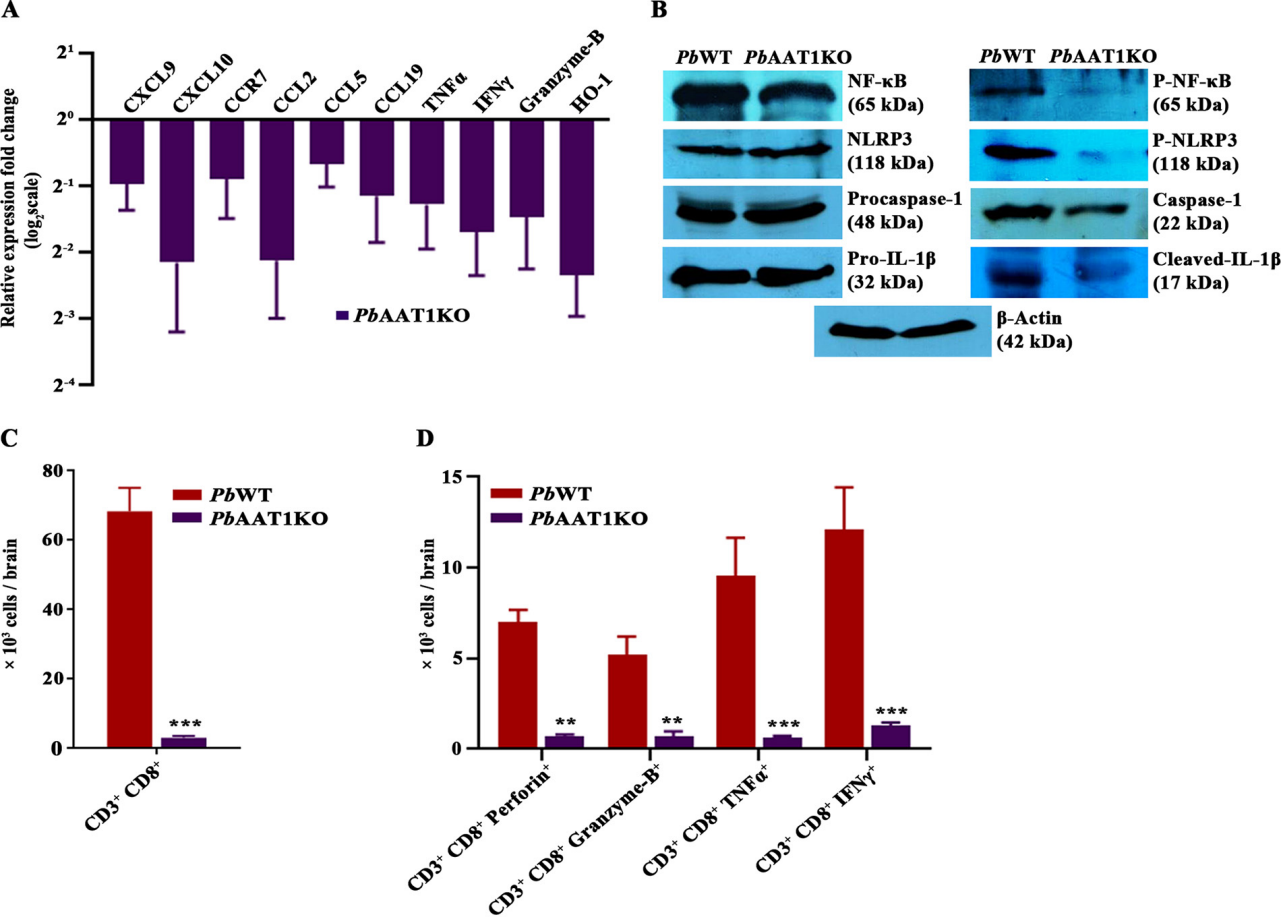
Brain inflammation and T cell infiltration in *Pb*AAT1KO^Luc^-infected mice. (A) RT-PCR analysis of cytokine and chemokine expression in brain RNA samples of *Pb*WT- and *Pb*AAT1KO-infected mice. The data (mean ± SEM) represent three different mice. (B) Western blotting analysis of phosphorylated NF-κB, phosphorylated NLRP3, cleaved caspase-1, and cleaved IL-1β levels in brain samples of *Pb*WT- and *Pb*AAT1KO-infected mice; 250 μg of total protein from the pooled brain homogenates of three different mice was used. (C) CD3^+^CD8^+^ cells in the brains of *Pb*WT- and *Pb*AAT1KO-infected mice. (D) CD3^+^CD8^+^ cells expressing perforin, granzyme B, TNF-α, and IFN-γ in the brains of *Pb*WT- and *Pb*AAT1KO-infected mice. The flow cytometry data (mean ± SEM) represent three different mice; ***, *P < *0.001; **, *P < *0.01. Data were analyzed by two-way ANOVA.

RT-PCR analysis performed for *Pb*AAT1KO-infected mouse brain samples showed a greater than 1.5-fold reduction in RNA levels of *CXCL9*, *CXCL10*, *CCR7*, *CCL2*, *CCL5*, *CCL19*, *TNF-α*, *IFN-γ*, *granzyme B*, and *HO-1* compared with *Pb*WT-infected mouse brain samples (Fig. 7A). To assess neuronal inflammation, brain homogenates were prepared for mice infected with *Pb*WT and *Pb*AAT1KO parasites. Phospho-NF-κB is a key transcription factor that is essential for NLRP3 inflammasome activation. Western analyses of brain homogenates showed a decrease in the levels of phosphorylated NF-κB in mice infected with *Pb*AAT1KO parasites. However, the levels of NF-κB were found to be comparable between *Pb*WT- and *Pb*AAT1KO-infected mice. Similar results were also observed for phosphorylated NLRP3 that is critical for NLRP3 inflammasome activation. In addition, there was a decrease in cleaved caspase-1 and cleaved IL-1β, the downstream events of NLRP3 inflammasome activation (Fig. 7B). These results suggested reduced neuronal inflammation in *Pb*AAT1KO-infected mice. Analyses of T cell infiltration in the *Pb*AAT1KO-infected mouse brain showed a significant decrease in the levels of CD3^+^CD8^+^ cells (Fig. 7C) and CD3^+^CD8^+^ cells expressing perforin, granzyme B, TNF-α, and IFN-γ (Fig. 7D; Fig. S5). Further, the activated T cell population (CD3^+^CD8^+^CD69^+^ triple-positive cells) was found to be low in *Pb*AAT1KO-infected mouse spleens, while the memory B cell population (CD19^+^B220^+^ CD11c^+^ triple-positive cells) was significantly high (Fig. S6). All these findings again confirmed the reduced neuronal inflammation and cerebral pathogenesis in *Pb*AAT1KO-infected mice.

### Genetic complementation restores FV morphology and ECM mortality.

**FIG 8:**
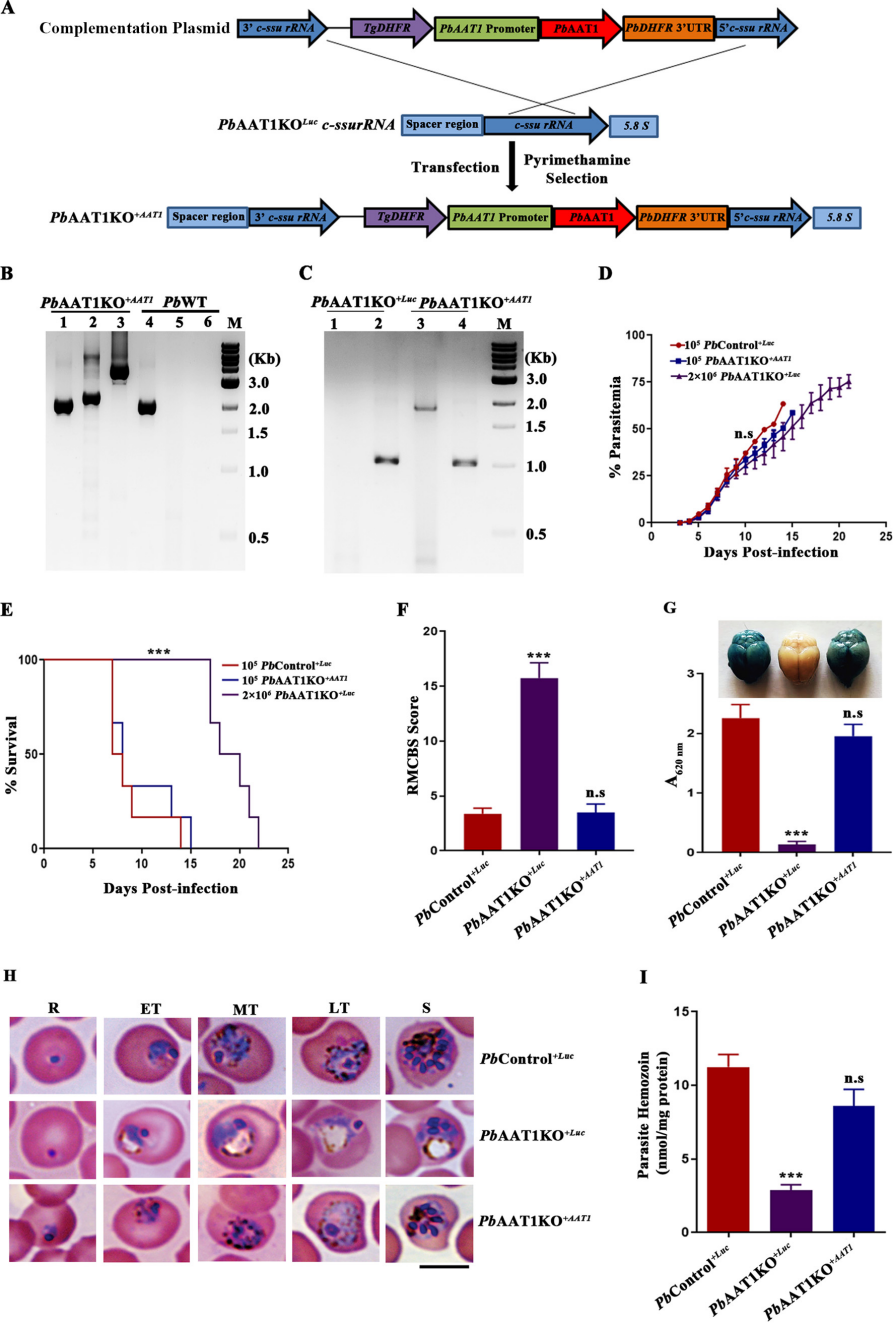
Genetic complementation of *AAT1* and restoration of cerebral pathogenesis, FV morphology, and Hz levels in *Pb*AAT1KO parasites. (A) Schematic representation of the recombination strategy followed to generate *Pb*AAT1KO*^+AAT1^* parasites. (B) Genomic DNA PCR confirmation of site-specific integration and *AAT1* complementation in *Pb*AAT1KO^Luc^ parasites; lanes 1 and 4, amplification of *AAT1* product (1.93 kb); lanes 2 and 5, integration at the *c-ssurRNA* locus confirmed by amplification using L665 (*Pb*DHFR 3′-UTR-specific) and L740 (5.8 S-specific) primers (2.16 kb); lanes 3 and 6, integration at the *c-ssurRNA* locus confirmed by amplification using the *Pb*AAT1 internal forward and L740 primers (3.38 kb); lane M, 1-kb ladder. (C) RT-PCR confirmation of *AAT1* complementation in *Pb*AAT1KO^Luc^ parasites; lanes 1 and 3, amplification of *AAT1* (1.84 kb); lanes 2 and 4, *GAPDH* control (1.01 kb); lane M, 1-kb ladder. (D) Growth analysis of *Pb*Control^+^^Luc^ (*n* = 6), *Pb*AAT1KO*^+AAT1^* (*n* = 6), and *Pb*AAT1KO*^+^*^Luc^ (*n* = 6) parasites in C57BL/6 mice; 10^5^ parasites were used to initiate *Pb*Control^+Luc^ and *Pb*AAT1KO*^+AAT1^* infections, and 2 × 10^6^ parasites were used to initiate *Pb*AAT1KO*^+^*^Luc^ infections. The data (mean ± SD) represent two different batches; n.s, not significant. Data were analyzed by two-way ANOVA. (E) Mortality curves of mice infected with *PbControl^+^*^Luc^, *Pb*AAT1KO*^+AAT1^*, and *Pb*AAT1KO*^+^*^Luc^ parasites. The data represent the mice used for growth curve analysis; ***, *P* < 0.001. Data were analyzed by log-rank (Mantel-Cox) test. (F) RMCBS score for mice infected with *Pb*Control^+^^Luc^ (*n* = 8), *Pb*AAT1KO*^+AAT1^* (*n* = 8), and *Pb*AAT1KO*^+^*^Luc^ (*n* = 8) parasites on days 7 and 8 postinfecion. *Pb*Control^+^^Luc^ data represent the mice that succumbed to ECM; n.s, not significant; ***, *P < *0.001. Data were analyzed by two-way ANOVA. (G) Evans blue extravasation and quantification of Evans blue in the brain samples of mice infected with *Pb*Control^+^^Luc^ (*n* = 3), *Pb*AAT1KO*^+AAT1^* (*n* = 3), and *Pb*AAT1KO*^+^*^Luc^ (*n* = 3) parasites on days 7 and 8 postinfection; n.s, not significant; ***, *P* < 0.001. Data were analyzed by two-way ANOVA. (H) Giemsa-stained images of peripheral blood smears prepared from mice infected with *Pb*Control^+^^Luc^, *Pb*AAT1KO*^+AAT1^*, and *Pb*AAT1KO*^+^*^Luc^ parasites. Images were captured using a 100× lens objective; scale bar = 5 μm. (I) Hz levels in *Pb*Control^+^^Luc^, *Pb*AAT1KO^+^^Luc^, and *Pb*AAT1KO^+AAT1^ parasites isolated from infected C57BL/6 mice (*n* = 6) on days 7 and 8 postinfection. The data represent (mean ± SEM) five mice in each group; n.s, not significant; ***, *P* < 0.001. Data were analyzed by two-way ANOVA.

Our next interest was to examine whether the reintroduction of AAT1 in *Pb*AAT1KO parasites could restore FV morphology and ECM mortality. For this, we treated *Pb*AAT1KO^Luc^ parasites with 5-fluorocytosine and removed the pyrimethamine-selectable hDHFR-yFCU selection cassette. The successful removal of hDHFR-yFCU was verified by the absence of mCherry fluorescence. The resultant parasites were transfected with *pL1102* plasmid, wherein the *Pbeef1α* promoter and the downstream red fluorescent protein (RFP) gene were replaced with the native *Pb*AAT1 promoter of 878 bp and the *AAT1* gene (Fig. 8A). Site-specific integration in the *ssurRNA* locus of *Pb*AAT1KO parasites and the successful complementation of *AAT1* in *Pb*AAT1KO^Luc^ parasites (*Pb*AAT1KO*^+AAT1^*) were verified by genomic DNA and RT-PCR analyses (Fig. 8B and C). Growth analysis studies showed that the growth of *Pb*AAT1KO*^+AAT1^* asexual-stage parasites was comparable with *Pb*WT parasites (Fig. 8D). Further, unlike *Pb*AAT1KO^Luc^-infected mice, more than 60% of mice infected with *Pb*AAT1KO*^+AAT1^* asexual-stage parasites died of ECM within day 10, when blood parasitemia was around 20% (Fig. 8E). The RMCBS score of *Pb*AAT1KO*^+AAT1^*-infected mice was less (Fig. 8F) with detectable Evans blue extravasation (Fig. 8G), confirming the ECM phenotype due to the loss of BBB integrity. H&E staining and immunofluorescence analyses of brain sections also showed cerebral pathogenesis (Fig. S7A and B). More importantly, Giemsa-stained and brightfield images of *Pb*AAT1KO*^+AAT1^* asexual-stage parasites showed the absence of swollen FVs with FV morphology similar to that of control parasites (Fig. 8H; Fig. S7C). There was also a restoration of Hz levels in *Pb*AAT1KO*^+AAT1^* parasites (Fig. 8I). The results obtained from genetic complementation further confirmed the association of AAT1 deletion with the swollen FV phenotype, less Hz formation, and ECM protection that were observed in *Pb*AAT1KO parasites.

### Sexual- and liver-stage development of *Pb*AAT1KO parasites.

**FIG 9:**
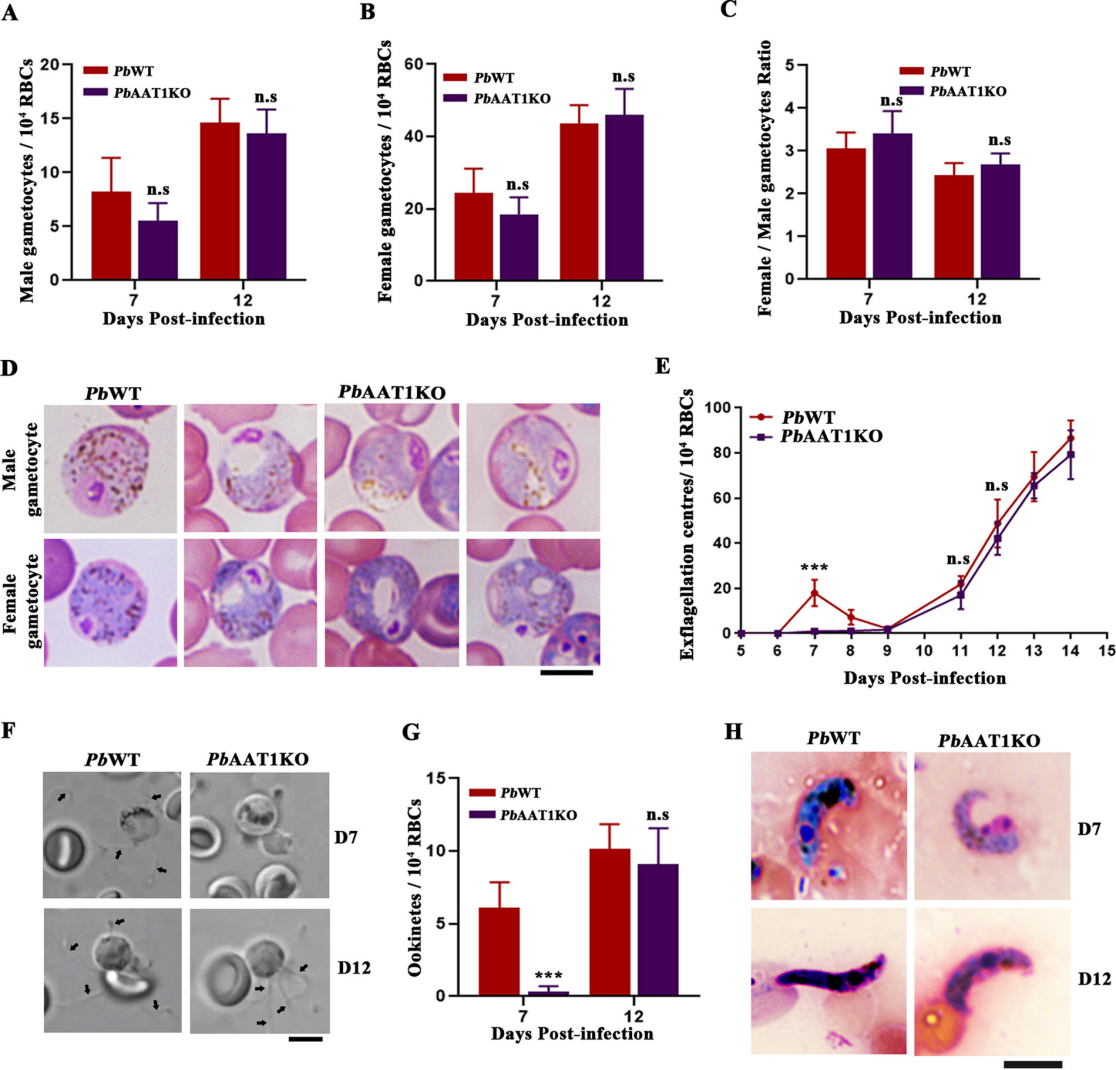
Gametocyte numbers, *in vitro* exflagellation, and ookinete formation in *Pb*AAT1KO parasites. (A) Number of male gametocytes counted from Giemsa-stained images of peripheral blood smears prepared from *Pb*WT- (*n* = 10) and *Pb*AAT1KO-infected (*n* = 10) mice. (B) Number of female gametocytes. The data (mean ± SD) represent three independent batches; n.s, not significant. Data were analyzed by two-tailed paired *t* test. (C) Female:male gametocyte ratio of *Pb*WT (*n* = 10) and *Pb*AAT1KO (*n* = 10) parasites. The data (mean ± SEM) represent three independent batches; n.s, not significant. Data were analyzed by two-sided paired *t* test. (D) Representative images showing the morphology of male and female gametocytes. Images were captured using a 100× lens objective; scale bar = 5 μm. (E) Number of exflagellation centers observed in *in vitro* exflagellation assays. The data (mean ± SD) represent 10 mice infected with *Pb*WT or *Pb*AAT1KO parasites from three independent batches; n.s, not significant; ***, *P < *0.001. Data were analyzed by two-way ANOVA. (F) Representative images of male gametocyte exflagellation. Images were captured using a 100× lens objective; scale bar = 5 μm. (G) Number of ookinetes formed in *in vitro* ookinete formation assays. The data (mean ± SEM) represent 10 mice infected with *Pb*WT or *Pb*AAT1KO parasites from three independent batches; n.s, not significant; ***, *P < *0.001. Data were analyzed by two-tailed paired *t* test. (H) Representative images of *in vitro* ookinetes. Images were captured using a 60× lens objective; scale bar = 5 μm.

Examination of male and female gametocyte numbers in the Giemsa-stained smears of *Pb*AAT1KO-infected mice on days 7 and 12 did not show significant differences (Fig. 9A and B), and the female and male gametocyte ratio (sex ratio) was comparable between *Pb*WT and *Pb*AAT1KO parasites (Fig. 9C). Swollen vacuoles were also detectable in the male and female gametocytes of *Pb*AAT1KO parasites (Fig. 9D). Interestingly, *in vitro* exflagellation analyses performed on infected blood samples showed hardly detectable exflagellation for *Pb*AAT1KO male gametocytes until day 9. However, exflagellation could be detected on day 11, and from day 11 onward, the number of exflagellation centers observed for *Pb*AAT1KO male gametocytes was comparable with *Pb*WT (Fig. 9E and F). This was also confirmed by performing *in vitro* ookinete formation analysis using *Pb*WT- and *Pb*AAT1KO-infected blood on days 7 and 12 postinfection. While there was an almost 94% decrease in the formation of *Pb*AAT1KO ookinetes on day 7, the ookinete numbers were comparable between *Pb*WT and *Pb*AAT1KO on day 12 (Fig. 9G and H). These results suggest a delay in the maturation of *Pb*AAT1KO male gametocytes.

**FIG 10:**
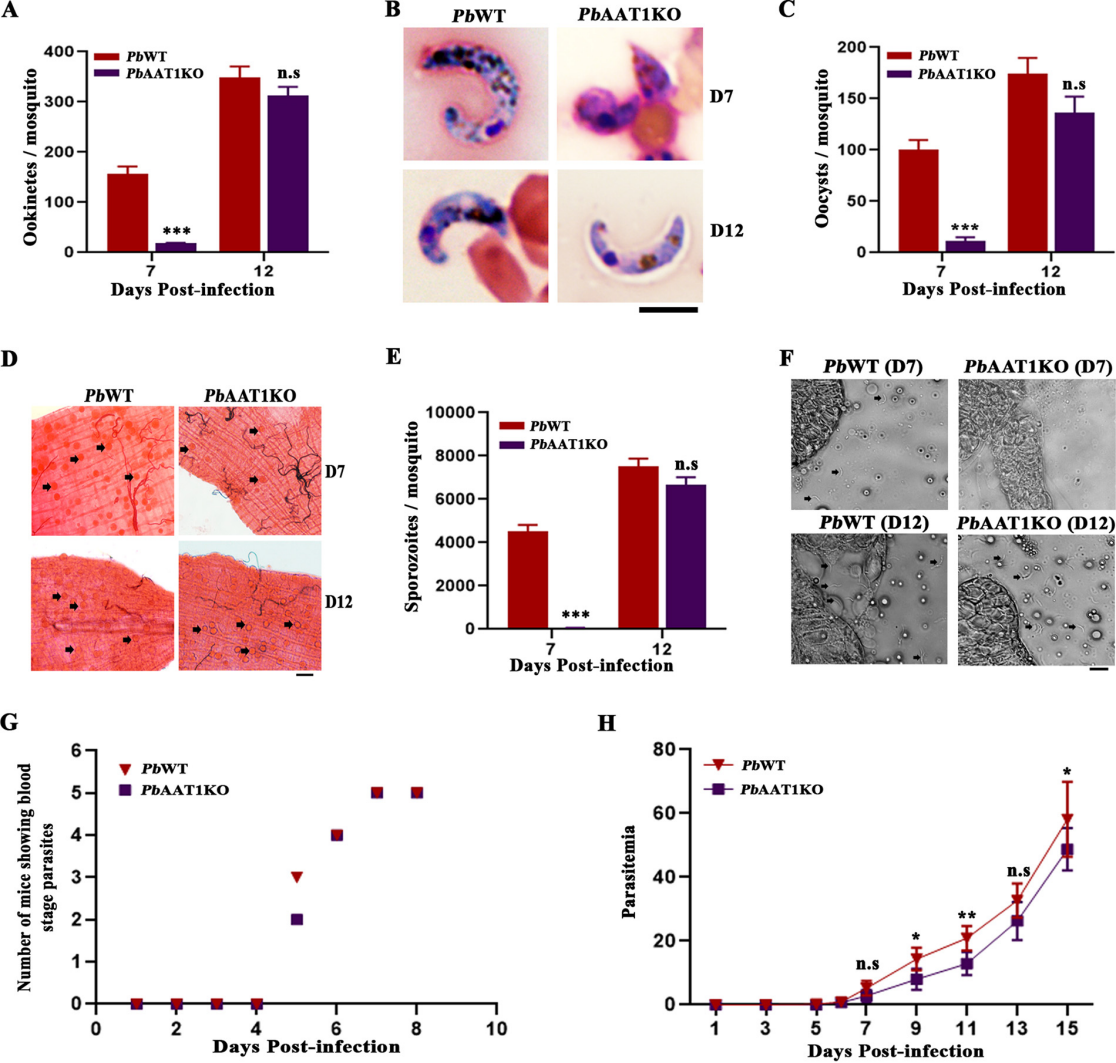
*In vivo* sexual- and liver-stage development of *Pb*AAT1KO parasites. (A) Number of ookinetes present in the blood bolus collected from A. stephensi mosquitoes 21 h postfeeding. For both ookinetes and oocysts, 20 mosquitoes were used. (B) Representative ookinete images. Images were captured using a 100× lens objective; scale bar = 5 μm. (C) Number of oocysts present in the A. stephensi guts dissected on day 10 postfeeding. The data (mean ± SEM) represent 20 mosquitoes from three independent batches; n.s, not significant; ***, *P < *0.001. Data were analyzed by two-tailed unpaired *t* test. For both ookinetes and oocysts, 20 mosquitoes were used. (D) Representative images of mosquito guts. Oocysts were stained using 0.05% mercurochrome. Black arrows represent oocysts appearing as red cysts. Images were captured using a 10× lens objective; scale bar = 50 μm. (E) Number of sporozoites present in the A. stephensi salivary glands dissected on day 19 postfeeding. The data (mean ± SEM) represent 30 mosquitoes from three independent batches; n.s, not significant; ***, *P < *0.001. Data were analyzed by two-tailed unpaired *t* test. (F) Representative images of mosquito salivary glands. Black arrows represent sporozoites. Images were captured using a 20× lens objective; scale bar = 20 μm. (G) Appearance of blood-stage parasites in naive C57BL/6 mice infected with *Pb*WT and *Pb*AAT1KO sporozoites. The numbers of mice showing detectable blood-stage parasites on the respective days postinfection are shown. (H) Growth analysis performed for mice infected with sporozoites to monitor the appearance of blood-stage parasites. Giemsa-stained smears were prepared from the peripheral blood for the respective days. The data (mean ± SD) represent five independent mice; n.s, not significant; *, *P* < 0.05; **, *P < *0.01. Data were analyzed by two-way ANOVA.

The results obtained from *in vitro* studies were further verified by performing *in vivo* infection studies in Anopheles stephensi mosquitoes by allowing them to take a blood meal from *Pb*AAT1KO-infected mice on days 7 and 12 postinfection. The dissection of day-7-fed infected mosquito guts 21 h after feeding suggested an almost 90% decrease in ookinetes compared with *Pb*WT (Fig. 10A and B). However, feeding mosquitoes with *Pb*AAT1KO-infected mice on day 12 postinfection did not show a significant decrease in the formation of ookinetes. While there was a significant increase in the proportions of *Pb*AAT1KO retorts formed *in vitro* and *in vivo* on day 7 postinfection, the proportions were comparable on day 12 postinfection between *Pb*WT and *Pb*AAT1KO infections (Fig. S8A and B). The decrease in the number of ookinetes formed in the day-7-fed mosquitoes was further reflected in the number of oocysts observed in the infected mosquito guts dissected on day 10 (Fig. 10C and D) and the number of sporozoites present in the salivary glands of the mosquitoes dissected on day 19 (Fig. 10E and F). No such changes were observed in the number of oocysts and sporozoites for day-12-fed *Pb*AAT1KO-infected mosquitoes (Fig. 10C to F). We next examined the effect of *AAT1* deletion on the liver-stage development of P. berghei by injecting 500 *Pb*AAT1KO sporozoites intravenously into C57BL/6 naive mice. There were no significant differences in the prepatent period and the appearance of asexual-stage parasites in the infected mouse blood between *Pb*WT and *Pb*AAT1KO sporozoite infections. As observed for the blood-stage propagations, *Pb*AAT1KO asexual parasites derived from the sporozoite infections showed slow growth in blood-stage development (Fig. 10G and H). All these findings suggest that *AAT1* deletion did not affect the sexual- and liver-stage development of P. berghei except for the delay observed in the male gametocyte exflagellation.

## DISCUSSION

Membrane transporters play a pivotal role in the life cycle of the malaria parasite. Approximately 3% of the parasite genome encodes membrane transporters, and these include various ion channels, primary active transporters, such as P-type ATPases, F-type ATPases, V-type ATPases, and ATP-binding cassette transporters, and secondary active transporters, such as the major facilitator superfamily, orphan transporters, etc. (37). The lack of key biosynthetic pathways for amino acids and purine nucleotides and the dependence of parasites on the host for various nutrients further emphasize the important role played by parasite transporters (38). High-throughput reverse genetic screening studies performed in P. falciparum and P. berghei suggested that more than two-thirds of transporters present a detectable phenotype in the life cycle stages of the malaria parasite (39). In this study, we have assessed the phenotype of AAT1KO using P. berghei as an *in vivo* rodent parasite model. Targeted deletion of FV-localized AAT1 in P. berghei did not affect asexual-stage parasite development, albeit, there was a slight delay in parasite growth and mortality due to anemia. Nevertheless, the assessment of ECM in C57BL/6 mice infected with AAT1KO parasites showed protection from cerebral pathogenesis. ECM protection was also observed when mice were infected with higher numbers of *Pb*AAT1KO parasites to match their growth with *Pb*WT parasites. These results suggested the association of *Pb*AAT1 with cerebral pathogenesis.

**FIG 11:**
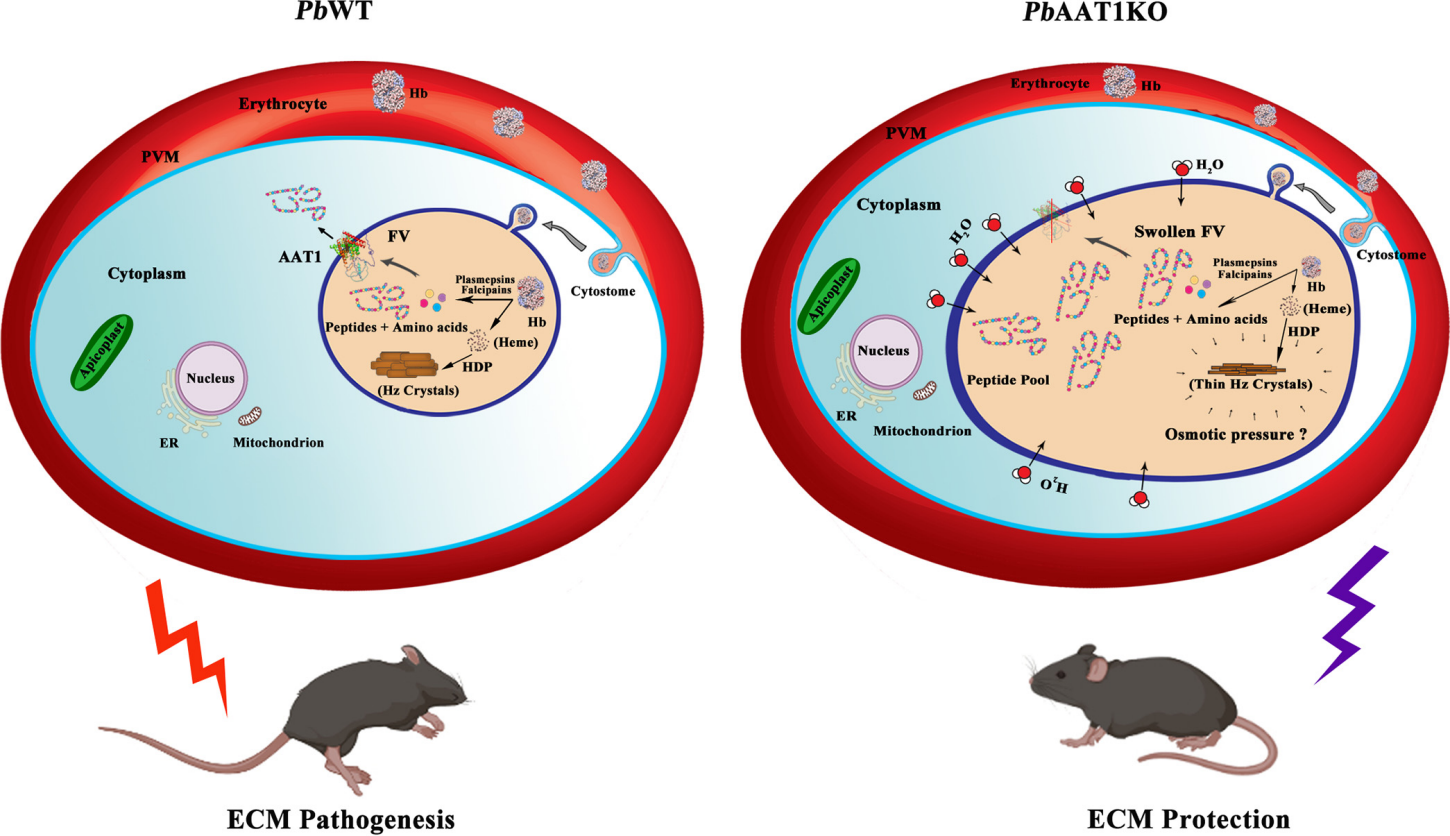
Model depicting the effect of *AAT1* deletion on P. berghei FVs. The release of peptides and amino acids from Hb degradation and the detoxification of Hb-heme into Hz are shown. The accumulation of Hb-derived peptides in *Pb*AAT1KO parasites leading to an increased osmotic pressure and a swollen FV phenotype with thin Hz crystal morphology are depicted. ECM protection observed in *Pb*AAT1KO-infected mice due to a compromised FV functionality is also represented; ER, endoplasmic reticulum; PVM, parasitophorous vacuolar membrane. BioRender was used to create the illustrations.

The FV localization of AAT1 and the direct association of Hz with disease severity together with the ECM protection phenotype observed in *Pb*AAT1KO-infected mice prompted us to examine FV morphology and Hz formation in *Pb*AAT1KO asexual parasites. Interestingly, *Pb*AAT1KO parasites displayed swollen FVs with the accumulation of Hb-derived peptides that were unique for *Pb*AAT1KO FVs and undetectable in *Pb*WT FVs. The total spectral intensity of these unique peptides represented ~75% of all the Hb-derived peptides identified in *Pb*AAT1KO FVs. The accumulation of Hb-derived peptides might increase the osmotic pressure within FVs, causing water influx leading to a swollen phenotype (Fig. 11). Further, at least 13 peptides identified from *Pb*WT FVs could not be detected in *Pb*AAT1KO FVs. These findings suggested a possible impairment of Hb digestion in *Pb*AAT1KO FVs besides the accumulation of Hb-derived peptides. Until now, the physiological function of parasitic AAT1 has remained unclear. The heterologous expression of *Pf*AAT1 in yeast cells lacking tryptophan biosynthesis caused quinoline hypersensitivity that could be suppressed by the exogenous addition of tryptophan (40). Another study expressing *Pf*AAT1 in Xenopus laevis oocytes could not detect significant changes in the uptake of radiolabeled amino acids (41). In agreement with the latter study, we did not find significant changes in amino acid levels of *Pb*AAT1KO FVs. Altogether, our results suggested that *Pb*AAT1 might transport Hb-derived peptides from the FV lumen to the parasite cytosol. A similar swollen FV phenotype has been reported for amantadine- and blasticidin-selected P. falciparum parasites carrying mutations in CRT and for P. falciparum parasites with reduced expression of CRT (31). Numerous factors, including pH, lipid composition, solubility of FV heme, integrity of FV, etc., might affect Hz formation and its morphology (42). It is also known that the size of Hz crystals varies between different *Plasmodium* species infecting humans, and their morphology seems to differ between mammalian and avian parasites (43). It was interesting to observe a thin morphology for the Hz crystals of *Pb*AAT1KO parasites with swollen FVs. It is not clear at this stage whether the thin morphology is due to mechanical stress exerted by the increased osmotic pressure in the swollen FVs or whether there are other reasons intrinsic to Hz formation (Fig. 11). Further, it would be of interest to examine whether the mutant CRT P. falciparum lines with swollen FVs also display a thin Hz morphology. Another important aspect of parasite AAT1 is its possible association with drug resistance. Based on the mutations identified in P. falciparum parasites, *Pf*AAT1 has been proposed to be involved in the efflux of multiple drugs (34, 44). Our results suggested that *AAT1* deletion renders P. berghei parasites less sensitive to quinolines such as chloroquine and amodiaquine, leading to recrudescence in infected mice. The swollen FV phenotype and reduced Hz formation might impair the optimal antimalarial activity of quinolines in *Pb*AAT1KO parasites, leading to the escape of a small proportion of parasites that can eventually cause recrudescence. Further studies are required to address the association of AAT1 with antimalarial activity and drug resistance.

Since *Pb*AAT1KO asexual-stage parasites synthesized less Hz, *Pb*AAT1KO-infected mice showed reduced Hz accumulation in the organs with fewer numbers of PMNs containing phagocytosed Hz. Hz is a proinflammatory PAMP that induces NLRP3 inflammasome activation and IL-1β production. Hz impairs the function of monocytes, macrophages, and dendritic cells, thereby compromising the innate immune response (9). Further, Hz restricts memory B cell and long-lived plasma cell responses and compromises humoral and cellular immunity. The circulating levels of phagocytosed Hz and Hz accumulation correlate strongly with malaria pathogenesis and the inflammation observed in the brain, placenta, and lungs (9, 45). In agreement with less Hz formation, mice infected with *Pb*AAT1KO parasites showed reduced systemic and neuronal inflammation and did not succumb to ECM. The plasma levels of proinflammatory cytokines IL-2, IL-6, TNF-α, and IFN-γ were significantly low, and those of anti-inflammatory cytokines IL-10 and IL-13 were significantly high. IL-2 levels are known to be upregulated in malaria infection, and IL-2 treatment can induce cerebral pathogenesis in ECM-resistant mice (46). It has also been shown that Hz can induce IL-6 and TNF-α expression (47). Serum IL-6 levels are highly elevated in children with CM, and IL-6 can contribute to CM pathogenesis (48). TNF-α plays an important role in CM by facilitating parasite sequestration through the induction of ICAM-1 in endothelial cells (49). Similarly, IFN-γ plays a prominent role in CM, and IFN-γ- and IFN-γ-receptor-deficient mice are protected from CM. IFN-γ can directly influence the function of endothelial cells, neurons, and astrocytes by inducing the expression of adhesion molecules, cytokines, and nitric oxide, thereby leading to BBB disruption and neurodysfunction (50, 51). Further, IFN-γ can facilitate transendothelial migration of CD4^+^ T cells that in turn mediate CD8^+^ T cell recruitment and activation, contributing to cerebral pathogenesis (52, 53). In contrast, the elevated levels of IL-10 and IL-13 are shown to alleviate ECM pathogenesis in mice (54).

*Pb*AAT1KO-infected mice showed a significant decrease (>1.5-fold) in the transcript levels of chemokines, chemokine receptors, cytokines, and cytotoxic effector molecules, such as *CXCL9*, *CXCL10*, *CCR7*, *CCL2*, *CCL5*, *CCL19*, *TNF-α*, *IFN-γ*, and *granzyme B*, which facilitate leukocyte migration, inflammation, and apoptosis. Many of them are known to be induced by Hz, and their levels are elevated in severe CM. There was a decrease in the expression of *HO-1* that is induced in neuronal inflammation. There was also a reduced infiltration of CD3^+^CD8^+^ T cells expressing perforin, granzyme B, TNF-α, and IFN-γ and decreased protein levels of phospho-NF-κB, phospho-NLRP3, cleaved caspase-1, and cleaved IL-1β in the brains of *Pb*AAT1KO-infected mice. All these results pointed to an overall decrease in the cellular and molecular signatures that mediate inflammation, endothelial and neuronal damage, and neuronal apoptosis.

Another important finding is the prominent delay observed in the exflagellation of *Pb*AAT1KO male gametocytes. *Pb*WT male gametocytes could exflagellate on day 7 and become transmissible through mosquitoes, leading to successful sporozoite formation. In contrast, *Pb*AAT1KO male gametocytes hardly showed exflagellation on day 7, with negligible transmission and almost undetectable sporozoites. Nevertheless, the exflagellation and transmission potential were comparable between *Pb*WT and *Pb*AAT1KO parasites on day 12. Male gametocyte maturation and exflagellation involve various molecular and cellular events, including regulation of transcription and translation, metabolic changes, replication, and axoneme assembly that are tightly associated with amino acid requirement (55). Further, Hb degradation plays an important role in the initial stages of gametocyte development. It is also possible that female gametocyte maturation is compromised in *Pb*AAT1KO parasites. Further detailed studies are required to understand the involvement of AAT1 in gametocyte maturation. Beyond gametocyte maturation, *Pb*AAT1KO parasites did not show significant differences in mosquito and liver-stage development, suggesting that the AAT1KO phenotype is restricted mainly to the asexual and gametocyte stages that involve Hb degradation. In summary, our study has shed new insights on yet another FV transporter and its association with FV functionality, Hz formation, disease pathogenesis, and gametocyte development. Our findings warrant detailed studies on the frequency and distribution of mutations in AAT1 of clinical isolates to understand its contribution toward antimalarial activity and resistance.

## MATERIALS AND METHODS

### Bioinformatics analyses.

AAT1 protein sequences of P. berghei, P. yoelii, P. falciparum, *P. reichenowi*, *P. ovale*, and P. vivax were retrieved from the PlasmoDB database. The following are the PlasmoDB accession numbers: PBANKA_1128300 (*Pb*AAT1), PY17X_1129800 (*Py*AAT1), PF3D7_0629500 (*Pf*AAT1), PRCDC_0627900 (*Pr*AAT1), PocGH01_11027800 (*Po*AAT1), and PVP01_1120000 (*Pv*AAT1). Multiple sequence alignment was performed using Clustal Omega (1.2.4). The PROTTER (version 1.0) model was used to predict the transmembrane domain topology and the number of transmembrane domains. Hydropathy, amphipathicity, secondary structure, and transmembrane topology of the AAT1 protein were predicted by the WHAT tool using JNET and MEMSAT programs (56).

### Animal ethics statement.

The experimental procedures performed in mice were approved by the Institutional Animal Ethics Committee (ILS/IAEC-57-AH/JAN-16) in accordance with the national guidelines of The Committee for the Purpose of Control and Supervision of Experiments on Animals (CPCSEA).

### Propagation of P. berghei asexual-stage parasites in mice.

BALB/c and ECM-susceptible C57BL/6 male/female mice of 7 to 8 weeks old weighing around 22 to 25 g were used. The mice were bred under clean and standard conditions with a temperature of 23 ± 4°C, a relative humidity of 30 to 70%, and a diurnal lighting cycle (12 h light/12 h dark) with *ad libitum* access to pellet food and water at the animal house facility of the Institute of Life Sciences, Bhubaneswar. Before the experiments, mice were acclimatized to the experimental environment for at least 5 days. Cryopreserved P. berghei parasites were thawed and passaged intraperitoneally in naive BALB/c or C57BL/6 mice. For initiating the blood-stage experiments, mice were intraperitoneally injected with 10^5^ or 2 × 10^6^ parasitized RBCs. Blood parasitemia was monitored by preparing Giemsa-stained thin blood smears prepared from the tail vein blood of infected mice and examining them under a light microscope.

### Generation of *Pb*WT*^+GFP-AAT1^* parasites.

*pL0027* plasmid containing a GFP-Luc-expressing cassette and *Tg*DHFR-TS as a selectable marker was used to generate *Pb*WT*^+GFP-AAT1^* transgenic parasites by replacing luciferase in the plasmid with AAT1. For this, *Pb*AAT1 was amplified from *Pb*WT genomic DNA using Phusion DNA polymerase with the following forward and reverse primers, respectively: 5′-GCCAGGATCCTGATGGCTAATAATATAGATATATTAGATTATTGTC-3′ and 5′-GCCCTCTAGACTAGTATATTATAATAAATGCGGTAACTATAGATG-3′. BamHI and XbaI restriction sites are underlined. The PCR product was digested with the respective enzymes and cloned into *pL0027* plasmid digested with BamHI and XbaI to excise the luciferase sequence. The recombinant plasmid was then linearized by digesting with ApaI and SacII, and the transfection was performed in *Pb*WT parasites with 4D-Nucleofector using P5 Primary Cell 4D-Nucleofector reagent (Lonza, V4XP-5012). The presence of *Tg*DHFR-TS selection marker allowed the selection of transgenic parasites (*Pb*WT*^+GFP-AAT1^*) using pyrimethamine (70 μg/mL) via drinking water, and clonal selection was performed by limiting dilution.

### Localization of *Pb*AAT1.

Immunofluorescence analysis of AAT1 localization in the asexual-stage parasites was performed as previously described (57). BALB/c mice were infected intraperitoneally with 10^5^ *Pb*WT*^+GFP^*^−^*^AAT1^* parasites, and on day 6 postinfection when parasitemia reached around 4 to 5%, 50 μL of infected mouse blood was collected, washed three times in phosphate-buffered saline (PBS), and overlaid on poly-l-lysine-coated coverslips for 1 h. After washing with PBS, the parasitized RBCs overlaid on coverslips were fixed with 4% paraformaldehyde in PBS containing 0.0075% glutaraldehyde for 30 min at room temperature. The parasitized RBCs were then permeabilized with 0.1% Triton X-100 (vol/vol) in PBS for 10 min and subsequently treated with 0.1 M glycine for 10 min. After blocking with 2% bovine serum albumin (BSA [wt/vol]) for 4 h at room temperature, the fixed parasitized RBCs were incubated with rabbit polyclonal anti-GFP antibody (Abcam, ab290; 1:500 dilution) for 5 h at room temperature. The cells were then washed with PBS, followed by treatment with rhodamine-conjugated donkey anti-rabbit secondary antibodies (Santa Cruz, 1:250 dilution) for 3 h at room temperature and treatment with 4′,6-diamidino-2-phenylindole (DAPI; 1 μg/mL in PBS) for 20 min. The coverslips were then dried and mounted on glass slides with ProLong Gold antifade (Invitrogen). Fluorescence images were acquired using an inverted Olympus IX83 microscope with a DP73 high-performance camera, and images were processed using Olympus cellSens Dimension software.

### Generation of *Pb*AAT1KO and *Pb*AAT1KO^Luc^ parasites.

*Pb*AAT1KO parasites were generated by transfecting *Pb*WT parasites with *pL0006* plasmid with 5′ and 3′ UTRs of the *AAT1* gene on either side of the human DHFR selectable marker using the double-crossover homologous recombination strategy, as previously described (58). *Pb*WT genomic DNA was used to amplify a 699-bp 5′ UTR of *Pb*AAT1 using Phusion DNA polymerase (Thermo) with forward (5′-GCCAGGGCCCTCCATTGCTGCTGTATTTTTATTCTG-3′) and reverse (5′-GCCCAGATCTTCTATTAAAAAATAGATGCAATCATTCATACC-3′) primers. Similarly, a 724-bp 3′ UTR of *Pb*AAT1 was amplified with forward (5′-GCCAGGTACCTGAAAGACCGAAGTGTGCTTTTTACTTTATAC-3′) and reverse (5′-GCCCGCGGCCGCGTGCAGTTTATAAGCCGAGCTTG-3′) primers. The restriction sites are underlined, and the resultant 5′-UTR and 3′-UTR fragments were digested with ApaI and BglII and KpnI and NotI, respectively. Digested fragments were then cloned into *pL0006* plasmid flanking the human DHFR expression cassette. The recombinant plasmid was then linearized with ApaI and NotI and transfected into purified mature schizonts of *Pb*WT. The transfected parasites were injected intravenously into 7- to 8-week-old naive BALB/c mice and selected using pyrimethamine, and clonal selection was done by limiting dilution. GFP-Luc-expressing *Pb*AAT1KO (*Pb*AAT1KO^Luc^) transgenic parasites were generated using GOMO-GFP-Luc plasmid with a GFP-Luc-expressing cassette with mCherry and a drug selection cassette consisting of human DHFR fused with yeast cytosine deaminase-uridyl phosphoribosyl transferase (hDHFR-yFCU) flanked on either side by 5′ and 3′ UTRs of the *Pb*AAT1 gene. The primers used to amplify the 5′ and 3′ UTRs were identical to those used for *Pb*AAT1KO parasites except for the restriction sites. The 5′-UTR and 3′-UTR primers had the restriction sites of SacII and NotI and XhoI and KpnI, respectively. Transfection and limiting dilutions were performed as described for *Pb*AAT1KO parasites. *Pb*Control^Luc^ parasites were generated by replacing the small subunit rRNA (*ssurRNA*) in *Pb*WT parasites with a GFP-Luc-expressing cassette containing mCherry using GOMO-GFP-Luc plasmid (59).

### Genetic complementation of *AAT1* in *Pb*AAT1KO^Luc^ parasites.

To perform genetic complementation of *AAT1*, *Pb*AAT1KO^Luc^-infected mice were treated with 5-fluorocytosine to remove the pyrimethamine-selectable hDHFR-yFCU gene. The successful generation of selectable marker-free parasites was confirmed by the loss of mCherry. The RFP gene under the control of the *Pb*eef1α promoter in *pL1102* plasmid was then replaced with the *Pb*AAT1 native promoter along with its coding sequence. To amplify the *Pb*AAT1 promoter and its coding sequence of 2.8 kb, the following forward and reverse primers were used: 5′-GCAACTTAAGTGTTATTTAATATGGTGCTTCAAAGTACT-3′ and 5′-GCAATCTAGACTAGTATATTATAATAAATGCGGTAACTATAGA-3′. AflII and XbaI restriction sites used for cloning are underlined. *Pb*AAT1KO*^+AAT1^* parasites with *AAT1* reintroduced in the *c-ssu-rRNA* locus were generated by transfecting the plasmid into *Pb*AAT1KO^Luc^ parasites, followed by pyrimethamine selection and limiting dilution.

### Quantitative assessment of ECM.

ECM manifestation in C57BL/6 mice infected with *Pb*WT, *Pb*AAT1KO, or *Pb*AAT1KO^Luc^ parasites was quantified using the rapid murine coma and behavior scale (RMCBS) (60). The scoring system uses 10 parameters to assess intracerebral pathology and neurologic impairment in mice by examining gait, balance, motor performance, body position, limb strength, touch escape, pinna reflex, toe pinch, aggression, and grooming. Each parameter was scored from 0 to 2 for a cumulative score of 0 to 20. The integrity of the blood-brain barrier (BBB) was assessed by Evans blue dye extravasation as previously described (61). In brief, the infected C57BL/6 mice were observed for the onset of clinical symptoms of ECM, such as convulsions, paraplegia, and ataxia, on day 7 postinfection. Mice were then intravenously injected with 200 μL of 2% Evans blue dye (Sigma-Aldrich) prepared in PBS. One hour postinjection, mice were transcardially perfused with PBS for 5 min, and brain samples were dissected to examine the extravasation of dye. The extent of vascular damage was determined by incubating brain samples in 2 mL of formamide (Merck) for 48 h at 37°C to extract Evans blue dye from the tissue and measuring the absorbance at 620 nm.

### Isolation of FVs.

FVs released during the schizont rupture of *Pb*WT and *Pb*AAT1KO parasites were isolated from the culture supernatant as described previously (59, 62). In brief, C57BL/6 mice infected with *Pb*WT or *Pb*AAT1KO parasites were sacrificed when blood parasitemia reached around 15 to 20%. Infected blood was collected at the late trophozoite stage on the previous night around 22:00 h, centrifuged at 1,000 × *g* for 5 min, and washed twice with RPMI 1640 containing 10% fetal bovine serum (FBS) to remove the plasma and buffy coat. Parasitized RBCs were then resuspended in 10 volumes of RPMI 1640 containing 10% FBS followed by overnight incubation at 37°C in a CO_2_ incubator. Giemsa-stained smears were prepared to monitor the maturation of schizonts, and FVs were isolated from the parasite cultures the next morning around 09:00 h. For this, the cultures were centrifuged two to three times at 200 × *g* for 5 min to remove the RBCs. The supernatant collected was centrifuged at 400 × *g* for 10 min to pellet the FVs devoid of merozoites. The FV pellets were then washed twice with 1 mL of PBS by centrifuging at 3,000 × *g* for 3 min. The purity of intact FVs was examined under brightfield using an inverted Olympus IX83 microscope with a DP73 high-performance camera and a 100× lens objective, followed by storage at −20°C.

### Transmission electron microscopy (TEM).

TEM analysis of FV morphology was performed as previously described with slight modifications (63). Briefly, *Pb*WT- and *Pb*AAT1KO-infected RBCs were washed with sodium cacodylate buffer (pH 7.2) and fixed for 24 h in a fixative solution containing 2.5% glutaraldehyde and 5 mM calcium chloride in 0.1 M sodium cacodylate (pH 7.2) at 4°C. Samples were then fixed for 1 h in 1% osmium tetroxide in cacodylate buffer. After rinsing with distilled water, samples were stained with 1% aqueous uranyl acetate for 1 h at room temperature. Thereafter, samples were dehydrated sequentially with increasing concentrations of ethanol (50, 70, 95, and 100%) and infiltrated with an epon/propylene oxide series in ratios of 1:1, 2:1, 3:1, and 4:1 for 6 h each. Samples were infiltrated overnight in EPON resin, followed by embedding and polymerization at 60°C for 48 h. Ultrathin sections (70 nm) of samples were prepared from embedded blocks using an ultramicrotome (Leica EM UC7) and were collected on copper grids (Ted Pella, Inc., formvar/carbon 200 mesh, copper). Sections were then stained with 2% aqueous uranyl acetate for 30 min and 1% lead citrate for 5 min, followed by imaging on a JEM-2100 Plus electron microscope operated at 200 kV with a lanthanum boride (LaB6) emitter. Digital images were acquired with a 3,000 × 3,000 16-bit pixel resolution and a magnification of ~12,000× using a complementary metal oxide semiconductor (CMOS) charge-coupled device (CCD) camera (point-point resolution of ~0.194 nm with the ultra-high resolution [UHR] objective lens) using Gatan’s Digital Micrograph (DM) software. FV and parasite areas (*A*_FV_ and *A*_P_) were measured using Image J software and expressed as the *A*_FV_/*A*_P_ ratio.

### Hz estimation.

Hz content of *Pb*WT and *Pb*AAT1KO parasites was estimated by following the protocol described earlier (59, 64). Briefly, the parasite pellets isolated by saponin lysis (0.15% saponin in PBS [wt/vol; Sigma-Aldrich, S4521]) were resuspended in 1 mL of sodium acetate buffer (100 mM, pH 5.0) and incubated overnight at 37°C with constant gentle shaking, followed by centrifugation at 10,000 × *g* for 5 min. The resultant pellets were washed with Tris buffer (100 mM, pH 8.0) containing 2.5% SDS by incubating at 37°C for 30 min, followed by alkaline bicarbonate (100 mM, pH 9.2) and distilled water. The final Hz pellet was resuspended in 100 mM NaOH containing 2.5% SDS, and the absorbance was read at 405 nm. Protein estimation was done using the Micro BCA protein assay kit (Thermo Scientific, 23235) for the supernatants. Hz content of *Pb*WT and *Pb*AAT1KO parasites was normalized against total protein and represented as nmol per mg of total protein. Hz content in various organs was estimated by homogenizing 50 mg of tissue in 50 mM Tris (pH 8.0) containing 50 mM NaCl, 5 mM CaCl_2_, and 1% Triton X-100, followed by incubation at 37°C for 12 h in the presence of proteinase K. The lysates were then processed to extract the Hz, as described, and the absorbance was measured at 405 nm for Hz pellets solubilized in 100 mM NaOH containing 2% SDS and 3 mM EDTA. Hz content in the organs normalized with respect to the parasite load was expressed as pmol per mg weight of the organ.

### Isolation of PMNs.

To assess the number of PMNs containing phagocytosed Hz, PMNs were isolated from the whole blood of *Pb*WT- and *Pb*AAT1KO-infected mice when parasitemia reached around 10%, as described previously (65) with slight modifications. Briefly, heparinized blood was collected from the infected mice, mixed with one-fifth volume of 4.5% dextran in isotonic solution (pH 7.4), and allowed to settle for 40 min at room temperature. RBC-depleted supernatant was then overlaid on 3 mL of Ficoll-Paque Plus (density of 1.077 g/mL; GE Healthcare) and centrifuged at 500 × *g* for 20 min at 20°C. The obtained pellet was resuspended in a lysis buffer (150 mM NH_4_Cl, 10 mM KHCO_3_, and 10 mM EDTA, pH 7.4) and left at room temperature for 10 min to remove residual RBCs. PMNs were pelleted by centrifuging at 100 × *g* for 10 min, washed twice, and resuspended in Hank’s balanced salt solution (HBSS). A thin smear of isolated PMNs was prepared for Giemsa staining to examine the number of PMNs containing phagocytosed Hz.

### Scanning electron microscopy analysis of Hz crystals.

*Pb*WT and *Pb*AAT1KO parasite pellets prepared by saponin lysis were used to isolate the Hz crystals. In brief, parasite pellets were resuspended in 10 volumes of 2.5% SDS and centrifuged at 14,000 × *g* for 10 min. After removing the supernatant and repeating the step once, the pellets were washed three times in 1 mL of distilled water. The purified Hz crystals were then analyzed by scanning electron microscopy as described previously with few modifications (66). In brief, Hz crystals were resuspended in 100 μL of distilled water, transferred onto round glass coverslips (12 mm), and dried for 24 h at 37°C. Samples were gold coated, mounted on a metallic sample holder, and analyzed using a field emission scanning electron microscope (JEOL JSM-IT800, Schottky) operated at a high vacuum with an accelerating voltage of 5 kV, standard probe current of 50, and working distance (WD) of 8 mm. Images were collected in secondary electron (SE) mode with different magnifications at a resolution of ~0.7 nm. The length, width, and thickness of Hz crystals from *Pb*WT and *Pb*AAT1KO parasites were measured using Image J software.

### Extraction and quantification of amino acids from the FVs.

Amino acids were extracted from the FV pellets of *Pb*WT and *Pb*AAT1KO parasites as described earlier with slight modifications (67). Briefly, 50 μL of FV pellets was resuspended in four volumes of 100% methanol and incubated at −80°C for 15 min, followed by centrifugation at 13,000 × *g* for 10 min at 4°C. The resulting supernatant was collected, and the pellets were then extracted twice by resuspending with 1 volume of 80% methanol in water (vol/vol), followed by sonication for 5 min in a water bath sonicator and centrifugation at 13,000 × *g* for 10 min at 4°C to collect the supernatants. All three supernatants were pooled, lyophilized, and stored at −80°C until LC-MS/MS and HPLC analysis. Quantification of amino acids in the FVs of *Pb*WT and *Pb*AAT1KO parasites was performed by HPLC and LC-MS/MS. For HPLC, the lyophilized FV extracts were solubilized with 50 μL of water. Samples (2.5 μL) were subjected to precolumn derivatization with o-phthalaldehyde (OPA) reagent (Agilent, 5061-3335) with the help of the automatic programmer, and amino acid separation was performed on a Poroshell 120 HPH-C_18_ column (4.6 mm × 100 mm × 2.7 μm) using an Agilent 1260 Infinity HPLC system, as per the manufacturer’s protocol (https://www.agilent.com/cs/library/applications/compendium-%20aminoacid-advancebio-5994-0033EN-us-agilent.pdf). Mobile phase consisted of: solvent A (Na_2_HPO_4_ [10 mM] and Na_2_B4O_7_ [10 mM], pH 8.2) and solvent B (CH_3_CN:CH_3_OH:H_2_O [45:45:10 vol/vol]). The identification of OPA-derivatized amino acids was done using a 1260 Infinity II fluorescence detector (excitation of 340 nm and emission of 450 nm). The amino acids were identified based on their retention time using the amino acid standards as reference. The relative peak areas of the amino acids present in *Pb*WT and *Pb*AAT1KO FVs were measured. The data were normalized with respect to the peak area obtained for norvaline. Amino acid quantification by LC-MS/MS was done using a Dionnex Ultimate3000 ultrahigh-performance liquid chromatography (UHPLC) system and an Acquity C_18_ column (1.8 μm, 2.1 mm × 100 mm) coupled with a Q Exactive mass spectrometer (Thermo Fisher Scientific). The lyophilized FV extracts were solubilized with 50 μL of water, derivatized with 6-aminoquinolyl-*N*-hydroxysuccinimidyl carbamate (AQC), and subsequently cleaned with solid phase extraction (SPE), followed by drying under vacuum. Samples were reconstituted in 50 μL of 0.5% acetonitrile and 0.1% formic acid, and 10 μL of the sample was used for HPLC with the following parameters: flow rate of 150 μL/min, autosampler temperature of 4°C, injection volume of 10 μL, and column temperature of 15°C using solvent A (10 mM ammonium acetate containing 0.1% formic acid) and solvent B (acetonitrile containing 0.1% formic acid) as mobile phase. The mass spectrometer was equipped with an electrospray ionization (ESI) source with an ESI spray voltage of 4,000/2,500 V, vaporizer temperature of 250°C, and nitrogen sheath gas and auxiliary gas flow rates of 30 and 10 arbitrary units, respectively. Acquisition was performed in parallel reaction monitoring mode at a resolution of 35,000 with a normalized collision energy of 25 eV. Calibration curves were plotted for the individual amino acids, and the samples were spiked with deuterated amino acids as internal standards. Relative changes in the levels of amino acids were calculated based on the peak areas of amino acids present in the FVs of *Pb*WT and *Pb*AAT1KO samples after normalizing with the peak area of the nonprotein amino acid citrulline.

### Peptidomic analysis of FVs.

Peptidomic analysis of *Pb*WT and *Pb*AAT1KO FVs was performed with pooled FV preparations by using the methanol extraction protocol as described previously with slight modifications (68). Briefly, peptides were serially extracted from FVs with different concentrations of methanol. For the first extraction, FV pellets were resuspended in 200 μL of 90% methanol in water (vol/vol) and incubated for 20 min at −80°C, followed by centrifugation at 13,000 × *g* for 10 min at 4°C to collect the supernatants. Pellets were then extracted twice with 200 μL of 80% methanol for 30 min on ice. For the second and third extractions, the suspensions were vortexed and briefly sonicated in a water bath sonicator for 1 min. The supernatants from all three extractions were pooled, lyophilized, and stored at −80°C. For LC-MS/MS analysis, the peptide samples were subjected to microflow reverse-phase LC in an Eksigent Ekspert Nano LC 425 system (SCIEX) coupled with a tandem quadrupole time-of-flight (TOF) SCIEX TripleTOF 5600^+^ ESI mass spectrometer. Samples were concentrated by SCIEX Micro Trap Cartridge (Chrome XP; C_18_-CL, 5-μm, 120-Å pore size), and the concentrated peptides were separated by using a SCIEX capillary reverse-phase column (Chrome XP, 3C_18_-CL-120, 3 μm, 120 Å, and 0.3 × 150 mm). The parameters and solvents used were a flow rate of 5 μL/min, solvent A (2% acetonitrile containing 0.1% formic acid [vol/vol] in HPLC-grade water), and solvent B (98% acetonitrile containing 0.1% formic acid [vol/vol]). The gradient program used for the separation was 2 to 50% solvent B for 28 min, followed by 50 to 90% solvent B for 1 min, 90% solvent B for 3 min, and 90 to 5% solvent B for 0.5 min with a final reequilibration with 2% solvent B for 2.5 min. Positive-ion and high-sensitivity modes with a full-scan resolution of 35,000 were used to record mass spectra and tandem mass spectra. The following ion source parameters were used: ion spray voltage floating (ISVF) = 5,500, ion source gas 1 (GS1) = 25, ion source gas 1 (GS2) = 22, and curtain gas flow (CUR) = 30. Nitrogen gas in a collision cell was used to fragment the precursor ions. TOF-MS and TOF-MS/MS spectra were calibrated with 100 fmol beta-galactosidase digest (SCIEX), and the peptide spectra and MS/MS spectra were recorded over mass/charge (*m*/*z*) ranges of 350 to 1,250 and 150 to 1,600, respectively, in data-dependent acquisition (DDA) mode. All the peptides identified were selected to calculate the total spectral intensities and MS2 counts of mouse Hb-derived peptides from *Pb*WT and *Pb*AAT1KO FVs. The spectral intensities and MS2 counts were normalized against the peptide intensities and MS2 counts of three parasite FV proteins, bergheilysin, GAPDH, and 31-kDa antigen. The relative fold changes of the spectral intensities and MS2 counts of mouse Hb-derived peptides that were common between *Pb*WT and *Pb*AAT1KO FVs were determined.

### H&E staining of brain sections.

*Pb*WT- and *Pb*AAT1KO-infected mice were perfused with cold PBS, and the brain samples were dissected and fixed in formalin for 72 h at room temperature. The samples were serially dehydrated with ethanol and treated with xylene for 1 h. Samples were then embedded in paraffin block, and tissue sections of 7-μm thickness were prepared using a Leica RM2125RT rotary microtome, placed on poly-l-lysine-coated glass slides, and dried at room temperature for 2 days. The sections were then treated with xylene and serially rehydrated with ethanol, followed by staining with H&E and analysis under a light microscope (59).

### Immunostaining of brain sections.

*Pb*WT- and *Pb*AAT1KO-infected mice were perfused sequentially with cold PBS and 4% paraformaldehyde in PBS. The brain samples were then dissected and fixed in 4% paraformaldehyde in PBS containing 20% sucrose for 24 h at 4°C, followed by incubation with 20% sucrose in PBS for 48 h and embedding on tissue-freezing medium (Leica Biosystems). Brain coronal sections of 30-μm thickness were prepared using a Leica CM1850 cryostat microtome, and heat-mediated antigen retrieval was performed by incubating sections at 95°C for 30 min in sodium citrate buffer (pH 9.0) (69). Sections were blocked with 1% BSA for 90 min and incubated with anti-CD31 mouse monoclonal antibody (Santa Cruz, sc-376764; 1:200 dilution) conjugated to Alexa Fluor 594 and anti-*Pb*GAPDH rabbit polyclonal serum (1:100 dilution) for 16 h at 4°C. Sections were then rinsed three times in wash buffer and incubated with fluorescein isothiocyanate (FITC)-conjugated donkey anti-rabbit IgG (Santa Cruz, sc-2090; 1:200 dilution) for 2 h at room temperature. After washing three times in wash buffer and counterstaining with DAPI (Sigma-Aldrich), the sections were dried overnight and mounted with ProLong Gold antifade reagent (Invitrogen). All fluorescence images were captured and analyzed using an Olympus IX83 microscope with a high-performance camera (DP73). For IgG extravasation, indirect immunoperoxidase staining for endogenous IgG was performed as previously described (69). In brief, the antigen-retrieved brain sections were treated with 3% H_2_O_2_ at room temperature for 30 min to block the endogenous peroxidase activity. The sections were then washed with PBS and incubated with horseradish peroxidase (HRP)-conjugated goat anti-mouse IgG (Abcam, ab97023; 1:250 dilution) in PBS containing 0.3% Triton X-100 and 0.1% BSA overnight at 4°C. After washing with PBS containing 0.1% Tween, the sections were incubated in ABC solution for 1.5 h at room temperature, as per the manufacturer’s instructions (Vector Labs, SK-4100), followed by diaminobenzidine tetrahydrochloride treatment for 5 min. The sections were thoroughly washed with distilled water to stop the reaction, counterstained with hematoxylin (HiMedia, S058) for 5 min at room temperature, ethanol dehydrated and xylene treated, and mounted with DPX mounting agent (Sigma-Aldrich). Brightfield images were captured using an Olympus IX83 microscope with a high-performance camera (DP73).

### Cytokine and chemokine analysis.

Plasma cytokine levels of *Pb*WT- and *Pb*AAT1KO-infected mice were estimated using a Bio-Plex Pro mouse cytokine Grp I panel 23-plex assay kit (Bio-Rad, M60009RDPD) according to the manufacturer’s instructions. The assay was performed on a Bio-Plex 200 system, and the data were analyzed using the Bio-Plex manager software. To analyze the transcript levels of cytokines, chemokines, and chemokine receptors, RNA isolated from the cold PBS-perfused brain samples of *Pb*WT- and *Pb*AAT1KO-infected mice was used. qPCRs were performed using a QuantiFast SYBR green RT-PCR kit (Qiagen, 204154) on a StepOne real-time PCR system (Applied Biosystems). The primers used are provided in Table S1 in the supplemental material. The expression levels were normalized against mouse *GAPDH*, and the fold changes in transcript levels of *Pb*AAT1KO-infected mice with respect to *Pb*WT-infected mice were determined using the ΔΔ*C_t_* method.

### *In vivo* and *ex vivo* bioluminescence imaging.

*In vivo* and *ex vivo* bioluminescence studies were performed using an IVIS Lumina XR imaging system, as described previously (53). Infected mice were injected intraperitoneally with 200 μL of d-luciferin substrate (Promega VivoGlo; 100 mg/kg dose) in PBS. After 5 min, mice were placed in the imaging chamber under anesthesia maintained at 2% isoflurane/0.2 L O_2_ per mouse per min using an XGI-8 gas anesthesia system. Whole-body bioluminescence images were acquired with medium binning, a 10-s exposure, and 12.5 field of view (FOV). For *ex vivo* imaging, the organs were dissected from the infected mice perfused with cold PBS. Quantitative analysis of bioluminescence signal from the whole body and organs was performed by measuring the luminescence signal intensity in photons per second using the region of interest (ROI) settings of the Living Image 3.0 software.

### Western blotting.

To examine the expression of GFP-AAT1 fusion protein, parasite pellets solubilized in 200 mM Tris (pH 6.8), 40% glycerol, and 2.5% SDS and incubated at 95°C for 10 min were subjected to SDS-PAGE, followed by Western blotting. Rabbit polyclonal anti-GFP antibody (ab290; 1:5,000 dilution) was used as the primary antibody. Western blotting of inflammatory parameters in the infected mouse brain was performed as described previously (59). In brief, the perfused brains of infected mice were homogenized in 50 mM Tris-Cl buffer (pH 7.5) containing 5 mM EDTA, 50 mM NaCl, 5 mM dithiothreitol (DTT), 0.1% NP-40, 50 mM NaF, 1 mM phenylmethylsulfonyl fluoride (PMSF), 1 mM Na_3_VO_4_, and 1× halt protease inhibitor cocktail (Thermo Fisher Scientific), followed by centrifugation at 18,000 × *g* for 20 min at 4°C. The resultant supernatant was collected, and the total protein concentration was estimated using a Micro BCA protein assay kit (Thermo Scientific, 23235). The following antibodies were used at a 1:1,000 dilution: anti-mouse NF-κΒ p65 (Invitrogen, 14-6731-81), anti-mouse phospho-NF-κB p65 (Ser536; Invitrogen, MA5-15160), anti-mouse NLRP3 (Invitrogen, PA5-20838), anti-mouse phospho-NLRP3 (Ser295; Invitrogen, PA5-105071), anti-mouse cleaved caspase-1 (Asp296; Cell Signaling Technology, 89332), and anti-GFP antibody (ab290). Anti-mouse IL-1β (Invitrogen, 701304), anti-mouse caspase 1 (Invitrogen, 14-9832-82), and anti-mouse β-actin (Cell Signaling Technology, 3700) were used at 1:250, 1:500, and 1:2,000 dilutions, respectively. Goat anti-rabbit IgG H&L (HRP; ab97051) and goat anti-mouse IgG H&L (HRP; ab97023) conjugated to HRP were used as secondary antibodies at a dilution of 1:10,000. The blots were developed using Pierce ECL Western blotting substrate (Thermo Fisher Scientific).

### Flow cytometry.

Flow cytometry analyses were performed as previously described (59, 70). The perfused brain samples were harvested and digested with RPMI 1640 containing 10% FBS, 2 U/mL DNase, and 0.05% collagenase D and passed through a 70-μm nylon cell strainer, followed by 5 min of incubation on ice. The resulting single-cell suspensions were centrifuged at 300 × *g* for 10 min at room temperature, and the pellet obtained was resuspended in RPMI 1640 containing 10% FBS and overlaid on a 30% Percoll gradient, followed by centrifugation at 400 × *g* for 20 min at room temperature. After treating the leukocyte pellet with RBC lysis buffer (155 mM NH_4_Cl, 10 mM NaHCO_3_, and 0.1 mM EDTA, pH 7.3) for 5 min at room temperature, the cells were stained for various extracellular and intracellular markers. The following fluorescent dye-conjugated antibodies were used for staining: anti-mouse CD3-FITC (clone 17A2, eBioscience), anti-mouse CD8a-APC-Cy7 (clone-53-6.7, BD Pharmingen), anti-mouse TNF-α-eFluor 450 (clone MP6-XT22, eBioscience), anti-mouse granzyme B-eFluor 450 (clone NGZB, eBioscience), anti-mouse IFN-γ-PE (clone XMG1.2, eBioscience), and anti-mouse perforin-PE (clone S16009A, BioLegend). For splenocyte isolation, perfused spleen samples were harvested, cut, and minced in 2 mg/mL collagenase D solution (5 mL/spleen, >0.15 U/mg) and incubated for 15 min at 37°C. Thereafter, tissue homogenates were passed through a 40-μm nylon mesh cell strainer. The cells were collected and centrifuged at 300 × *g* for 10 min at 4°C. The resulting pellet was resuspended in 2 mL of RBC lysis buffer and maintained for 4 min at room temperature. Finally, an equal volume of wash buffer (0.5% BSA and 2 mM EDTA in PBS) was added to the sample and centrifuged at 300 × *g* for 10 min at 4°C. The splenocyte pellet was washed twice with wash buffer and resuspended in 1 mL of fluorescence-activated cell sorting (FACS) buffer (3% BSA and 5 mM EDTA in PBS); 5 × 10^6^ cells/mL were used for FACS analysis. The fluorescent dye-conjugated antibodies used for staining were anti-mouse CD3-FITC (clone 17A2, eBioscience), anti-mouse CD4-PE-Cy7 (GK1.5, eBioscience), anti-mouse CD8-PerCP-Cy5.5 (clone 53–6.7, Thermo Fisher Scientific), anti-mouse CD69-eFluor 450, (FN50, eBioscience), CD19-PE (eBio1D3, eBioscience), anti-mouse-CD45R(B220)-PE-Vio770 (RA3-6B2, Miltenyi Biotec), and CD11c-APC (MJ4-27G12, Miltenyi Biotec). Single-fluorochrome staining was performed to compensate for the spectral overlap. Data were acquired on a BD LSRFortessa cytometer and were analyzed using FlowJo v10.6.1 software.

### *In vitro* exflagellation and ookinete formation assays.

*In vitro* exflagellation assays for male gametocytes were performed as described earlier (71). The exflagellation of male gametocytes was examined daily from days 5 to 14 postinfection by collecting 5 to 10 μL of the infected blood in heparinized exflagellation medium (RPMI 1640 containing 25 mM HEPES, 10% heat-inactivated FBS, 25 mM sodium bicarbonate, and 100 μM xanthurenic acid at pH 8) and incubating for 15 min at 19°C. Exflagellation centers were counted using a phase-contrast objective. A minimum of 50 to 60 fields were examined to confirm the absence of exflagellation centers. Live imaging of exflagellation was performed under brightfield with an Olympus IX83 inverted microscope using a 60× lens objective. *In vitro* ookinete assays were performed on days 7 and 12 postinfection using 10 μL of the infected mouse blood collected in exflagellation medium and incubated at 19°C for 21 h. Giemsa smears were prepared to examine the numbers of ookinetes and retorts formed.

### *In vivo* sexual- and liver-stage studies.

A. stephensi mosquitoes were bred and maintained under the standard insectary conditions of 27°C and 75 to 80% humidity with a diurnal lighting cycle of 12 h light/12 h dark at the Institute of Life Sciences, Bhubaneswar. For colony maintenance, adult female mosquitoes (5 to 7 days old) were blood fed on anesthetized BALB/c mice, and eggs were collected on damp filter paper that hatched within 2 to 3 days. Larvae and pupae were maintained as per the standard procedures, and adult mosquitoes were fed on 10% (wt/vol) sucrose solution containing 0.05% *para*-aminobenzoic acid (PABA) (72). For *in vivo* sexual-stage studies, A. stephensi mosquitoes starved for 24 h were fed on anesthetized *Pb*WT- or *Pb*AAT1KO-infected mice either on day 7 or day 12 postinfection when blood parasitemia was around ~10 or ~25%, respectively. After removing the unfed mosquitoes, fed mosquitoes were maintained at 19°C with 70 to 80% relative humidity. At 20 h postfeeding, mosquito guts were dissected to remove the blood bolus. Thin smears were prepared, and Giemsa staining was performed to analyze the morphology and the number of ookinetes and retorts formed. To examine the number of oocysts, mosquito guts were dissected on day 10 postfeeding, stained with 0.5% mercurochrome (Sigma-Aldrich) in water for 10 min at room temperature, washed several times with phosphate-buffered saline (PBS), and analyzed using a light microscope. For sporozoites, salivary glands were dissected on day 19 postfeeding, and the extracted sporozoites were quantified using a hemocytometer. Liver-stage infection was initiated by intravenously injecting 500 sporozoites into naive C57BL/6 mice. The prepatent period and appearance of blood-stage parasites were determined by examining the Giemsa-stained smears prepared from the tail vein blood collected from the infected mice.

### Other procedures.

Genomic DNA and RNA isolations and Southern blotting were performed using the standard protocols. *Pb*AAT1 3′-UTR digoxigenin (DIG)-labeled probe was prepared using a DIG DNA labeling kit (Roche, 11093657910). The hybridized DIG-labeled probe was detected using a DIG luminescent detection kit (Roche, 11363514910), as per the manufacturer’s protocol. For assessing the parasite load, qPCR analysis was performed using *Pb*GAPDH primers and genomic DNA prepared from the infected mouse blood. Chloroquine and amodiaquine were dissolved in water, and α,β-arteether was diluted with arachis oil. Chloroquine and amodiaquine were administered through the intraperitoneal route. For α,β-arteether, a single dose of 1 mg/mouse was administered through the intramuscular route on day 5 postinfection.

### Statistical analyses.

All graphs were plotted using GraphPad Prism 7.0 (GraphPad Software). Statistical analyses were performed using two-tailed, unpaired *t* tests and analysis of variance (ANOVA). The log-rank (Mantel-Cox) test was used for the survival analyses.

## References

[1] World Health Organization. 2022. World malaria report. https://www.who.int/teams/global-malaria-programme/reports/world-malaria-report-2022. Accessed 18 December 2022.

[2] Zimmerman GA, Castro-Faria-Neto H. 2010. Persistent cognitive impairment after cerebral malaria: models, mechanisms and adjunctive therapies. Expert Rev Anti Infect Ther 8:1209–1212. doi:10.1586/eri.10.117. 21073283

[3] Jagannathan P, Kakuru A. 2022. Malaria in 2022: increasing challenges, cautious optimism. Nat Commun 13:2678. doi:10.1038/s41467-022-30133-w. 35562368 PMC9106727

[4] Francis SE, Sullivan DJ, Jr, Goldberg AD. 1997. Hemoglobin metabolism in the malaria parasite *Plasmodium falciparum*. Annu Rev Microbiol 51:97–123. doi:10.1146/annurev.micro.51.1.97. 9343345

[5] Lew VL, Tiffert T, Ginsburg H. 2003. Excess hemoglobin digestion and the osmotic stability of *Plasmodium falciparum*-infected red blood cells. Blood 101:4189–4194. doi:10.1182/blood-2002-08-2654. 12531811

[6] Ridley RG, Dorn A, Vippagunta SR, Vennerstrom JL. 1997. Haematin (haem) polymerization and its inhibition by quinoline antimalarials. Ann Trop Med Parasitol 91:559–566. doi:10.1080/00034983.1997.11813174. 9329993

[7] Pham TT, Lamb TJ, Deroost K, Opdenakker G, Van den Steen PE. 2021. Hemozoin in malarial complications: more questions than answers. Trends Parasitol 37:226–239. doi:10.1016/j.pt.2020.09.016. 33223096

[8] Sullivan AD, Ittarat I, Meshnick SR. 1996. Patterns of haemozoin accumulation in tissue. Parasitology 112:285–294. doi:10.1017/S003118200006580X. 8728992

[9] Olivier M, Van Den Ham K, Shio MT, Kassa FA, Fougeray S. 2014. Malarial pigment hemozoin and the innate inflammatory response. Front Immunol 5:25. doi:10.3389/fimmu.2014.00025. 24550911 PMC3913902

[10] Parroche P, Lauw FN, Goutagny N, Latz E, Monks BG, Visintin A, Halmen KA, Lamphier M, Olivier M, Bartholomeu DC, Gazzinelli RT. 2007. Malaria hemozoin is immunologically inert but radically enhances innate responses by presenting malaria DNA to Toll-like receptor 9. Proc Natl Acad Sci USA 104:1919–1924. doi:10.1073/pnas.0608745104. 17261807 PMC1794278

[11] Gowda DC, Wu X. 2018. Parasite recognition and signaling mechanisms in innate immune responses to malaria. Front Immunol 9:3006. doi:10.3389/fimmu.2018.03006. 30619355 PMC6305727

[12] Tiemi Shio M, Eisenbarth SC, Savaria M, Vinet AF, Bellemare MJ, Harder KW, Sutterwala FS, Bohle DS, Descoteaux A, Flavell RA, Olivier M. 2009. Malarial hemozoin activates the NLRP3 inflammasome through Lyn and Syk kinases. PLoS Pathog 5:e1000559. doi:10.1371/journal.ppat.1000559. 19696895 PMC2722371

[13] Shio MT, Kassa FA, Bellemare MJ, Olivier M. 2010. Innate inflammatory response to the malarial pigment hemozoin. Microbes Infect 12:889–899. doi:10.1016/j.micinf.2010.07.001. 20637890

[14] Dasari P, Heber SD, Beisele M, Torzewski M, Reifenberg K, Orning C, Fries A, Zapf AL, Baumeister S, Lingelbach K, Udomsangpetch R. 2012. Digestive vacuole of *Plasmodium falciparum* released during erythrocyte rupture dually activates complement and coagulation. Blood 119:4301–4310. doi:10.1182/blood-2011-11-392134. 22403252

[15] Grau GE, Craig AG. 2012. Cerebral malaria pathogenesis: revisiting parasite and host contributions. Future Microbiol 7:291–302. doi:10.2217/fmb.11.155. 22324996

[16] Gazzinelli RT, Kalantari P, Fitzgerald KA, Golenbock DT. 2014. Innate sensing of malaria parasites. Nat Rev Immunol 14:744–757. doi:10.1038/nri3742. 25324127

[17] Chugh M, Sundararaman V, Kumar S, Reddy VS, Siddiqui WA, Stuart KD, Malhotra P. 2013. Protein complex directs hemoglobin-to-hemozoin formation in *Plasmodium falciparum*. Proc Natl Acad Sci USA 110:5392–5397. doi:10.1073/pnas.1218412110. 23471987 PMC3619337

[18] Saliba KJ, Kirk K. 1999. pH regulation in the intracellular malaria parasite, *Plasmodium falciparum*: H^+^ extrusion via a V-type H^+^-ATPase. J Biol Chem 274:33213–33219. doi:10.1074/jbc.274.47.33213. 10559194

[19] Papalexis V, Siomos MA, Campanale N, Guo XG, Kocak G, Foley M, Tilley L. 2001. Histidine-rich protein 2 of the malaria parasite, *Plasmodium falciparum*, is involved in detoxification of the by-products of haemoglobin degradation. Mol Biochemistry Parasitol 115:77–86. doi:10.1016/S0166-6851(01)00271-7. 11377742

[20] Matz JM, Drepper B, Blum TB, van Genderen E, Burrell A, Martin P, Stach T, Collinson LM, Abrahams JP, Matuschewski K, Blackman MJ. 2020. A lipocalin mediates unidirectional heme biomineralization in malaria parasites. Proc Natl Acad Sci USA 117:16546–16556. doi:10.1073/pnas.2001153117. 32601225 PMC7368307

[21] Jani D, Nagarkatti R, Beatty W, Angel R, Slebodnick C, Andersen J, Kumar S, Rathore D. 2008. HDP—a novel heme detoxification protein from the malaria parasite. PLoS Pathog 4:e1000053. doi:10.1371/journal.ppat.1000053. 18437218 PMC2291572

[22] Banerjee R, Liu J, Beatty W, Pelosof L, Klemba M, Goldberg DE. 2002. Four plasmepsins are active in the *Plasmodium falciparum* food vacuole, including a protease with an active-site histidine. Proc Natl Acad Sci USA 99:990–995. doi:10.1073/pnas.022630099. 11782538 PMC117418

[23] Sijwali PS, Rosenthal PJ. 2004. Gene disruption confirms a critical role for the cysteine protease falcipain-2 in hemoglobin hydrolysis by *Plasmodium falciparum*. Proc Natl Acad Sci USA 101:4384–4389. doi:10.1073/pnas.0307720101. 15070727 PMC384756

[24] Duffin KL, Goldberg DE. 1999. Identification and characterization of falcilysin, a metallopeptidase involved in hemoglobin catabolism within the malaria parasite *Plasmodium falciparum*. J Biol Chem 274:32411–32417. doi:10.1074/jbc.274.45.32411. 10542284

[25] Quevillon E, Spielmann T, Brahimi K, Chattopadhyay D, Yeramian E, Langsley G. 2003. The *Plasmodium falciparum* family of Rab GTPases. Gene 306:13–25. doi:10.1016/S0378-1119(03)00381-0. 12657463

[26] Lamarque M, Tastet C, Poncet J, Demettre E, Jouin P, Vial H, Dubremetz JF. 2008. Food vacuole proteome of the malarial parasite *Plasmodium falciparum*. Proteomics Clin Appl 2:1361–1374. doi:10.1002/prca.200700112. 21136929

[27] Dalal S, Klemba M. 2007. Roles for two aminopeptidases in vacuolar hemoglobin catabolism in *Plasmodium falciparum*. J Biol Chem 282:35978–35987. doi:10.1074/jbc.M703643200. 17895246

[28] Skinner-Adams TS, Stack CM, Trenholme KR, Brown CL, Grembecka J, Lowther J, Mucha A, Drag M, Kafarski P, McGowan S, Whisstock JC. 2010. *Plasmodium falciparum* neutral aminopeptidases: new targets for anti-malarials. Trends Biochem Sci 35:53–61. doi:10.1016/j.tibs.2009.08.004. 19796954

[29] Martin RE, Kirk K. 2004. The malaria parasite’s chloroquine resistance transporter is a member of the drug/metabolite transporter superfamily. Mol Biol Evol 21:1938–1949. doi:10.1093/molbev/msh205. 15240840

[30] Koenderink JB, Kavishe RA, Rijpma SR, Russel FG. 2010. The ABCs of multidrug resistance in malaria. Trends Parasitol 26:440–446. doi:10.1016/j.pt.2010.05.002. 20541973

[31] Pulcini S, Staines HM, Lee AH, Shafik SH, Bouyer G, Moore CM, Daley DA, Hoke MJ, Altenhofen LM, Painter HJ, Mu J. 2015. Mutations in the *Plasmodium falciparum* chloroquine resistance transporter, PfCRT, enlarge the parasite’s food vacuole and alter drug sensitivities. Sci Rep 5:14552. doi:10.1038/srep14552. 26420308 PMC4588581

[32] Ferreira PE, Holmgren G, Veiga MI, Uhlén P, Kaneko A, Gil JP. 2011. *Pf*MDR1: mechanisms of transport modulation by functional polymorphisms. PLoS One 6:e23875. doi:10.1371/journal.pone.0023875. 21912647 PMC3164660

[33] Shafik SH, Cobbold SA, Barkat K, Richards SN, Lancaster NS, Llinás M, Hogg SJ, Summers RL, McConville MJ, Martin RE. 2020. The natural function of the malaria parasite’s chloroquine resistance transporter. Nat Commun 11:3922. doi:10.1038/s41467-020-17781-6. 32764664 PMC7413254

[34] Cowell AN, Istvan ES, Lukens AK, Gomez-Lorenzo MG, Vanaerschot M, Sakata-Kato T, Flannery EL, Magistrado P, Owen E, Abraham M, LaMonte G. 2018. Mapping the malaria parasite druggable genome by using *in vitro* evolution and chemogenomics. Science 359:191–199. doi:10.1126/science.aan4472. 29326268 PMC5925756

[35] Wang Z, Cabrera M, Yang J, Yuan L, Gupta B, Liang X, Kemirembe K, Shrestha S, Brashear A, Li X, Porcella SF. 2016. Genome-wide association analysis identifies genetic loci associated with resistance to multiple antimalarials in *Plasmodium falciparum* from China-Myanmar border. Sci Rep 6:33891. doi:10.1038/srep33891. 27694982 PMC5046179

[36] Egan TJ, Ross DC, Adams PA. 1994. Quinoline anti-malarial drugs inhibit spontaneous formation of β-haematin (malaria pigment). FEBS Lett 352:54–57. doi:10.1016/0014-5793(94)00921-X. 7925942

[37] Martin RE, Ginsburg H, Kirk K. 2009. Membrane transport proteins of the malaria parasite. Mol Microbiol 74:519–528. doi:10.1111/j.1365-2958.2009.06863.x. 19796339

[38] Kenthirapalan S, Waters AP, Matuschewski K, Kooij TW. 2016. Functional profiles of orphan membrane transporters in the life cycle of the malaria parasite. Nat Commun 7:10519. doi:10.1038/ncomms10519. 26796412 PMC4736113

[39] Martin RE. 2020. The transportome of the malaria parasite. Biol Rev 95:305–332. doi:10.1111/brv.12565. 31701663

[40] Tindall SM, Vallières C, Lakhani DH, Islahudin F, Ting KN, Avery SV. 2018. Heterologous expression of a novel drug transporter from the malaria parasite alters resistance to quinoline antimalarials. Sci Rep 8:2464. doi:10.1038/s41598-018-20816-0. 29410428 PMC5802821

[41] Cobbold SA, Llinás M, Kirk K. 2016. Sequestration and metabolism of host cell arginine by the intraerythrocytic malaria parasite *Plasmodium falciparum*. Cell Microbiol 18:820–830. doi:10.1111/cmi.12552. 26633083

[42] Kapishnikov S, Hempelmann E, Elbaum M, Als-Nielsen J, Leiserowitz L. 2021. Malaria pigment crystals: the Achilles’ heel of the malaria parasite. ChemMedChem 16:1515–1532. doi:10.1002/cmdc.202000895. 33523575 PMC8252759

[43] Noland GS, Briones N, Sullivan DJ, Jr. 2003. The shape and size of hemozoin crystals distinguishes diverse *Plasmodium* species. Mol Biochem Parasitol 130:91–99. doi:10.1016/S0166-6851(03)00163-4. 12946845

[44] Amambua-Ngwa A, Button-Simons KA, Li X, Kumar S, Brenneman KV, Ferrari M, Checkley LA, Haile MT, Shoue DA, McDew-White M, Tindall SM. 2022. The amino acid transporter *Pf*AAT1 modulates chloroquine resistance and fitness in malaria parasites. bioRxiv. doi:10.1101/2022.05.26.493611. PMC10322710 37169919

[45] Deroost K, Lays N, Noppen S, Martens E, Opdenakker G, Van den Steen PE. 2012. Improved methods for haemozoin quantification in tissues yield organ-and parasite-specific information in malaria-infected mice. Malar J 11:166. doi:10.1186/1475-2875-11-166. 22583751 PMC3473299

[46] Sherry BA, Alava G, Tracey KJ, Martiney J, Cerami A, Slater AF. 1995. Malaria-specific metabolite hemozoin mediates the release of several potent endogenous pyrogens (TNF, MIP-1 alpha, and MIP-1 beta) *in vitro*, and altered thermoregulation *in vivo*. J Inflamm 45:85–96. doi:10.1016/S1995-7645(11)60220-4. 7583361

[47] Lyke KE, Burges R, Cissoko Y, Sangare L, Dao M, Diarra I, Kone A, Harley R, Plowe CV, Doumbo OK, Sztein MB. 2004. Serum levels of the proinflammatory cytokines interleukin-1 beta (IL-1β), IL-6, IL-8, IL-10, tumor necrosis factor alpha, and IL-12 (p70) in Malian children with severe *Plasmodium falciparum* malaria and matched uncomplicated malaria or healthy controls. Infect Immun 72:5630–5637. doi:10.1128/IAI.72.10.5630-5637.2004. 15385460 PMC517593

[48] Mandala WL, Msefula CL, Gondwe EN, Drayson MT, Molyneux ME, MacLennan CA. 2017. Cytokine profiles in Malawian children presenting with uncomplicated malaria, severe malarial anemia, and cerebral malaria. Clin Vaccine Immunol 24:e00533-16. doi:10.1128/CVI.00533-16. 28122790 PMC5382826

[49] Chakravorty SJ, Craig A. 2005. The role of ICAM-1 in *Plasmodium falciparum* cytoadherence. Eur J Cell Biol 84:15–27. doi:10.1016/j.ejcb.2004.09.002. 15724813

[50] Amani V, Vigário AM, Belnoue E, Marussig M, Fonseca L, Mazier D, Rénia L. 2000. Involvement of IFN-γ receptor-mediated signaling in pathology and anti-malarial immunity induced by *Plasmodium berghei* infection. Eur J Immunol 30:1646–1655. doi:10.1002/1521-4141(200006)30:6<1646::AID-IMMU1646>3.0.CO;2-0. 10898501

[51] Hunt NH, Golenser J, Chan-Ling T, Parekh S, Rae C, Potter S, Medana IM, Miu J, Ball HJ. 2006. Immunopathogenesis of cerebral malaria. Int J Parasitol 36:569–582. doi:10.1016/j.ijpara.2006.02.016. 16678181

[52] Belnoue E, Potter SM, Rosa DS, Mauduit M, Grüner AC, Kayibanda M, Mitchell AJ, Hunt NH, Renia L. 2008. Control of pathogenic CD8^+^ T cell migration to the brain by IFN-γ during experimental cerebral malaria. Parasite Immunol 30:544–553. doi:10.1111/j.1365-3024.2008.01053.x. 18665903

[53] Claser C, Malleret B, Gun SY, Wong AY, Chang ZW, Teo P, See PC, Howland SW, Ginhoux F, Rénia L. 2011. CD8^+^ T cells and IFN-γ mediate the time-dependent accumulation of infected red blood cells in deep organs during experimental cerebral malaria. PLoS One 6:e18720. doi:10.1371/journal.pone.0018720. 21494565 PMC3073989

[54] Chin VK, Chong WC, Haniza H, Basir R. 2021. Modulating effects of IL-4, IL-10 and IL-13 on the course of *Plasmodium berghei* malaria infection in mice. J Transl Med 24:92–105. doi:10.22452/jummec.vol24no2.13.

[55] Liu Z, Miao J, Cui L. 2011. Gametocytogenesis in malaria parasite: commitment, development and regulation. Future Microbiol 6:1351–1369. doi:10.2217/fmb.11.108. 22082293 PMC5711484

[56] Zhai Y, Saier MH. 2001. A web-based program (WHAT) for the simultaneous prediction of hydropathy, amphipathicity, secondary structure, and transmembrane topology for a single protein sequence. J Mol Microbiol Biotechnol 3:501–502. 11545267

[57] Tonkin CJ, van Dooren GG, Spurck TP, Struck NS, Good RT, Handman E, Cowman AF, McFadden GI. 2004. Localization of organellar proteins in *Plasmodium falciparum* using a novel set of transfection vectors and a new immunofluorescence fixation method. Mol Biochem Parasitol 137:13–21. doi:10.1016/j.molbiopara.2004.05.009. 15279947

[58] Braks JA, Franke-Fayard B, Kroeze H, Janse CJ, Waters AP. 2006. Development and application of a positive-negative selectable marker system for use in reverse genetics in *Plasmodium*. Nucleic Acids Res 34:e39. doi:10.1093/nar/gnj033. 16537837 PMC1401515

[59] Chandana M, Anand A, Ghosh S, Das R, Beura S, Jena S, Suryawanshi AR, Padmanaban G, Nagaraj VA. 2022. Malaria parasite heme biosynthesis promotes and griseofulvin protects against cerebral malaria in mice. Nat Commun 13:4028. doi:10.1038/s41467-022-31431-z. 35821013 PMC9276668

[60] Carroll RW, Wainwright MS, Kim KY, Kidambi T, Gomez ND, Taylor T, Haldar K. 2010. A rapid murine coma and behavior scale for quantitative assessment of murine cerebral malaria. PLoS One 5:e13124. doi:10.1371/journal.pone.0013124. 20957049 PMC2948515

[61] Pamplona A, Ferreira A, Balla J, Jeney V, Balla G, Epiphanio S, Chora Â, Rodrigues CD, Gregoire IP, Cunha-Rodrigues M, Portugal S. 2007. Heme oxygenase-1 and carbon monoxide suppress the pathogenesis of experimental cerebral malaria. Nat Med 13:703–710. doi:10.1038/nm1586. 17496899

[62] Dasari P, Reiss K, Lingelbach K, Baumeister S, Lucius R, Udomsangpetch R, Bhakdi SC, Bhakdi S. 2011. Digestive vacuoles of *Plasmodium falciparum* are selectively phagocytosed by and impair killing function of polymorphonuclear leukocytes. Blood 118:4946–4956. doi:10.1182/blood-2011-05-353920. 21911835

[63] Gomes-Santos CS, Braks J, Prudêncio M, Carret C, Gomes AR, Pain A, Feltwell T, Khan S, Waters A, Janse C, Mair GR. 2011. Transition of *Plasmodium* sporozoites into liver stage-like forms is regulated by the RNA binding protein Pumilio. PLoS Pathog 7:e1002046. doi:10.1371/journal.ppat.1002046. 21625527 PMC3098293

[64] Tripathi AK, Khan SI, Walker LA, Tekwani BL. 2004. Spectrophotometric determination of *de novo* hemozoin/β-hematin formation in an *in vitro* assay. Anal Biochem 325:85–91. doi:10.1016/j.ab.2003.10.016. 14715288

[65] Walev I, Tappe D, Gulbins E, Bhakdi S. 2000. Streptolysin O-permeabilized granulocytes shed l-selectin concomitantly with ceramide generation via neutral sphingomyelinase. J Leukoc Biol 68:865–872. doi:10.1189/jlb.68.6.865. 11129654

[66] Pisciotta JM, Scholl PF, Shuman JL, Shualev V, Sullivan DJ. 2017. Quantitative characterization of hemozoin in *Plasmodium berghei* and *vivax*. Int J Parasitol Drugs Drug Resist 7:110–119. doi:10.1016/j.ijpddr.2017.02.001. 28279945 PMC5342986

[67] Olszewski KL, Morrisey JM, Wilinski D, Burns JM, Vaidya AB, Rabinowitz JD, Llinás M. 2009. Host-parasite interactions revealed by *Plasmodium falciparum* metabolomics. Cell Host Microbe 5:191–199. doi:10.1016/j.chom.2009.01.004. 19218089 PMC2737466

[68] Lewis IA, Wacker M, Olszewski KL, Cobbold SA, Baska KS, Tan A, Ferdig MT, Llinás M. 2014. Metabolic QTL analysis links chloroquine resistance in *Plasmodium falciparum* to impaired hemoglobin catabolism. PLoS Genet 10:e1004085. doi:10.1371/journal.pgen.1004085. 24391526 PMC3879234

[69] Strangward P, Haley MJ, Shaw TN, Schwartz JM, Greig R, Mironov A, de Souza JB, Cruickshank SM, Craig AG, Milner DA, Jr, Allan SM. 2017. A quantitative brain map of experimental cerebral malaria pathology. PLoS Pathog 13:e1006267. doi:10.1371/journal.ppat.1006267. 28273147 PMC5358898

[70] Ryg-Cornejo V, Ioannidis LJ, Hansen DS. 2013. Isolation and analysis of brain-sequestered leukocytes from *Plasmodium berghei* ANKA-infected mice. J Vis Exp 71:50112. doi:10.3791/50112. PMC3582690 23329000

[71] Guttery DS, Poulin B, Ferguson DJ, Szöőr B, Wickstead B, Carroll PL, Ramakrishnan C, Brady D, Patzewitz EM, Straschil U, Solyakov L. 2012. A unique protein phosphatase with kelch-like domains (PPKL) in *Plasmodium* modulates ookinete differentiation, motility and invasion. PLoS Pathog 8:e1002948. doi:10.1371/journal.ppat.1002948. 23028336 PMC3447748

[72] MR4 staff. 2010. Methods in *Anopheles* Research. MR4, ATCC, National Institutes of Health, Centers for Disease Control and Prevention, Manassas, VA, Washington, DC, Atlanta, GA.

