# Supplementary material for: Significance of Plasmodium berghei Amino Acid Transporter 1 in Food Vacuole Functionality and Its Association with Cerebral Pathogenesis
Source: Microbiol Spectr. 2023 Mar 28;11(2):e04943-22. doi: 10.1128/spectrum.04943-22 (PMC10101031; doi:10.1128/spectrum.04943-22)
Supplement: Supplemental file 1 — Supplemental material. Download spectrum.04943-22-s0001.pdf, PDF file, 3.4 MB [file spectrum.04943-22-s0001.pdf]

Supplementary Information

Significance of *Plasmodium berghei* amino acid transporter 1 in the food vacuole functionality and its association with cerebral pathogenesis

Aditya Anand, Manjunatha Chandana, Sourav Ghosh, Rahul Das, Nalini Singh, Pradeed Mini Vaishalli, Nagavara Prasad Gantasala, Govindarajan Padmanaban and Viswanathan Arun Nagaraj

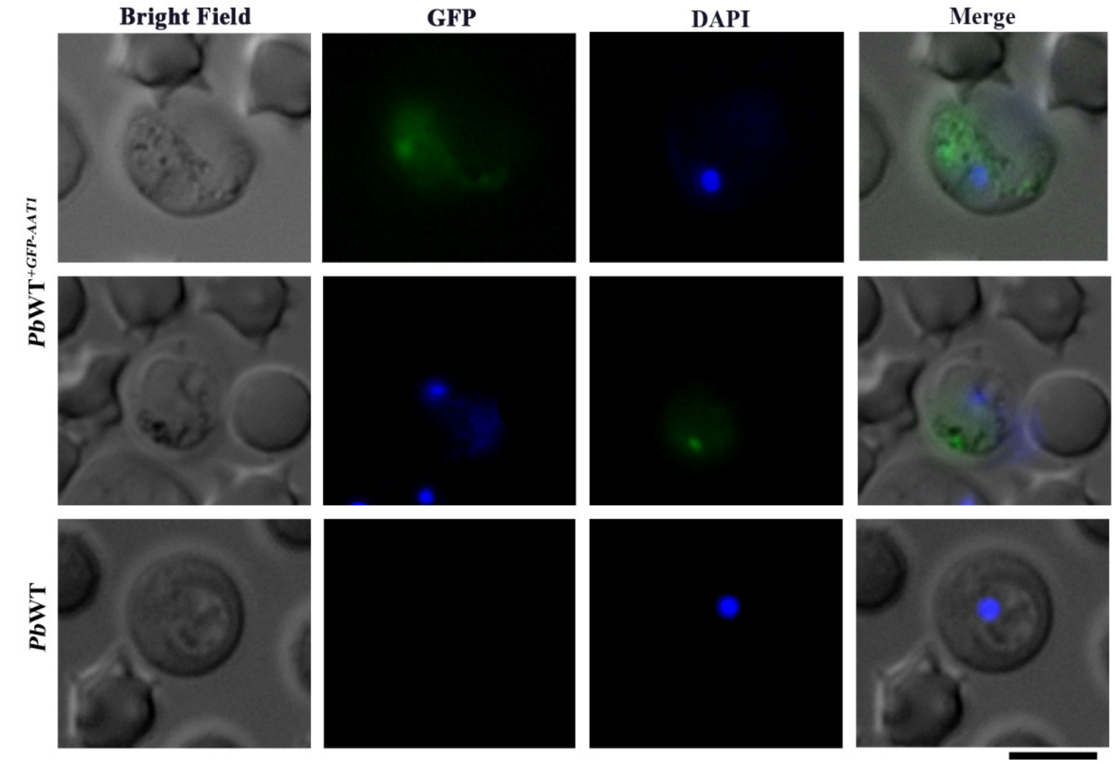

Fig. S1: Live fluorescence imaging of *Pb*WT$^{+GFP\text{-}AAT1}$ transgenic parasites. Live cell imaging of GFP fluorescence in *Pb*WT$^{+GFP\text{-}AAT1}$ parasites expressing GFP-tagged *Pb*AAT1. *Pb*WT parasites were used as control. DAPI was used for nuclear staining (blue). Images were captured using 100x objective lens. Scale bar = 5 µm.

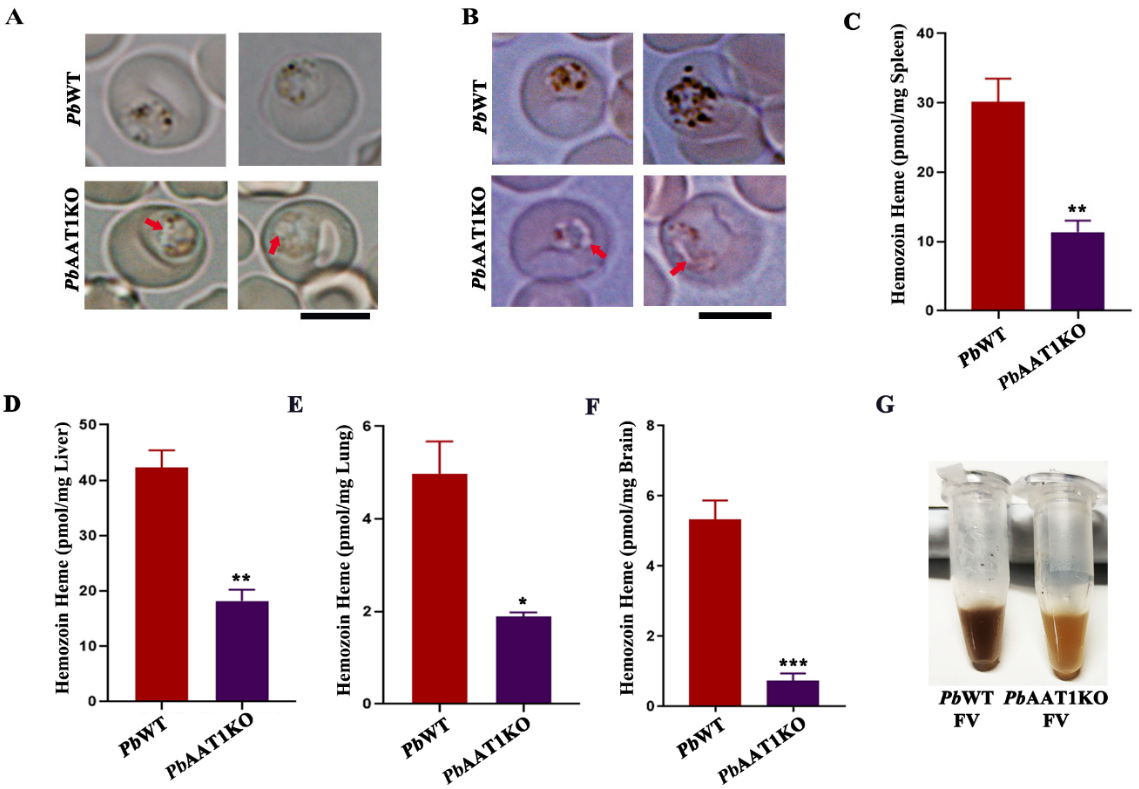

Fig. S2: Analyses of FVs in *Pb*AAT1KO parasites and Hz levels in the organs of *Pb*AAT1KO-infected mice. **(A)** Bright field images of live *Pb*AAT1KO-infected RBCs showing swollen FVs. **(B)** Bright field images of paraformaldehyde-fixed *Pb*AAT1KO-infected RBCs showing swollen FVs. Red arrows represent swollen FVs. Images were captured using 100x objective lens. Scale bar = 5 µm. **(C-F)** Quantification of parasite Hz in the spleen, liver, lungs and brain of mice *Pb*WT-(n=3) and *Pb*AAT1KO-infected mice (n=3). The data represent mean ± SEM (**P*<0.05; ***P*<0.01; ****P*<0.001; unpaired t-test; two-tailed). **(G)** Comparison of colour differences in the FV preparations of *Pb*WT and *Pb*AAT1KO parasites reflecting Hz levels.

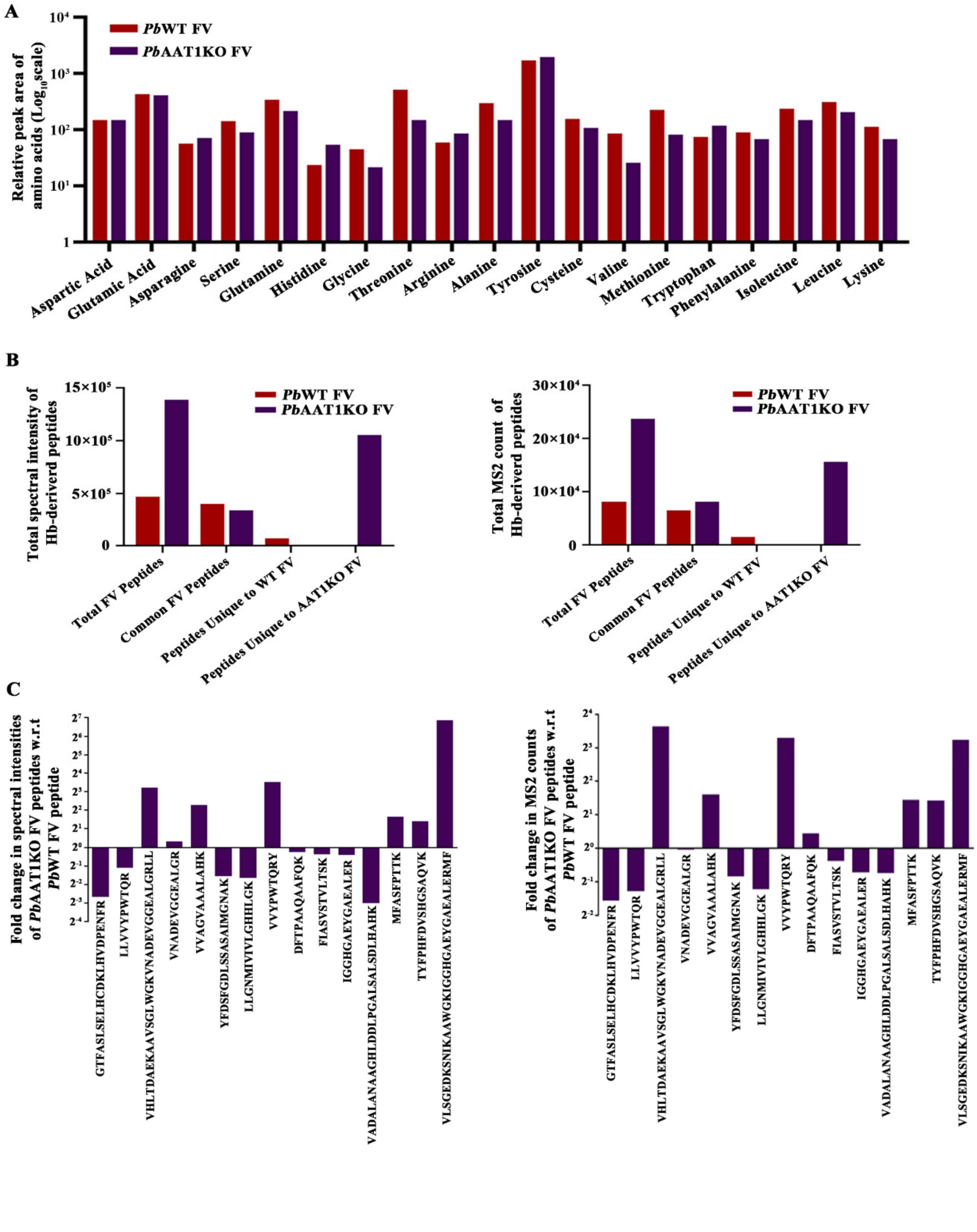

Fig. S3: HPLC analysis of amino acids, and spectral intensities of Hb-derived peptides in *Pb*AAT1KO FVs. (A) The relative peak areas of the amino acids present in *Pb*WT and

*Pb*AAT1KO FVs analysed by HPLC. The data represent two independent preparations. For each preparation, three FV pellets of *Pb*WT and *Pb*AAT1KO were pooled separately. The relative peak areas of the respective amino acids were normalized against norvaline. **(B)** Total spectral intensities and MS2 counts of total, common and unique Hb-derived peptides identified in the FVs of *Pb*WT and *Pb*AAT1KO parasites. The LC-MS/MS data correspond to the schematic represented in **Fig. 4D**. The peptide intensities and MS2 counts of mouse Hb-derived peptides were normalized against the peptide intensities and MS2 counts of parasite FV proteins - bergheilysin, GAPDH and 31 kDa antigen. **(C)** Fold changes in the spectral intensities and MS2 counts of Hb-derived peptides that were common between FVs of *Pb*WT and *Pb*AAT1KO parasite.

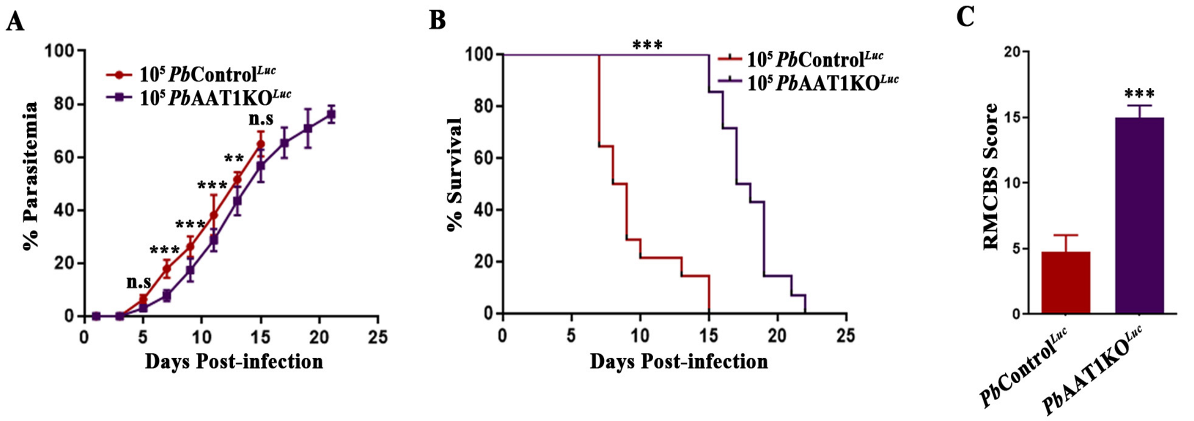

Fig. S4: Asexual stage phenotype of *Pb*AAT1KO^{Luc}-infected mice. (A) Growth curves of *Pb*Control^{Luc} (n=14) and *Pb*AAT1KO^{Luc} (n=14) parasites in C57BL/6 mice. 10^5 parasites were used to initiate *Pb*Control^{Luc} and *Pb*AAT1KO^{Luc} infections. The data (mean $\pm$ SD) represent three different batches (****P*<0.001, n.s - not significant; Two-way ANOVA). **(B)** Survival curves of C57BL/6 mice infected with *Pb*Control^{Luc} (n=14) and *Pb*AAT1KO^{Luc} (n=14) parasites (****P*<0.001; log-rank Mantel-Cox test). The data represent the mice used for parasite growth analysis. **(C)** RMCBS score for mice infected with *Pb*Control^{Luc} (n=8) and *Pb*AAT1KO^{Luc} (n=8) parasites. The data represent mean $\pm$ SD (****P*<0.001, unpaired t-test; two-tailed).

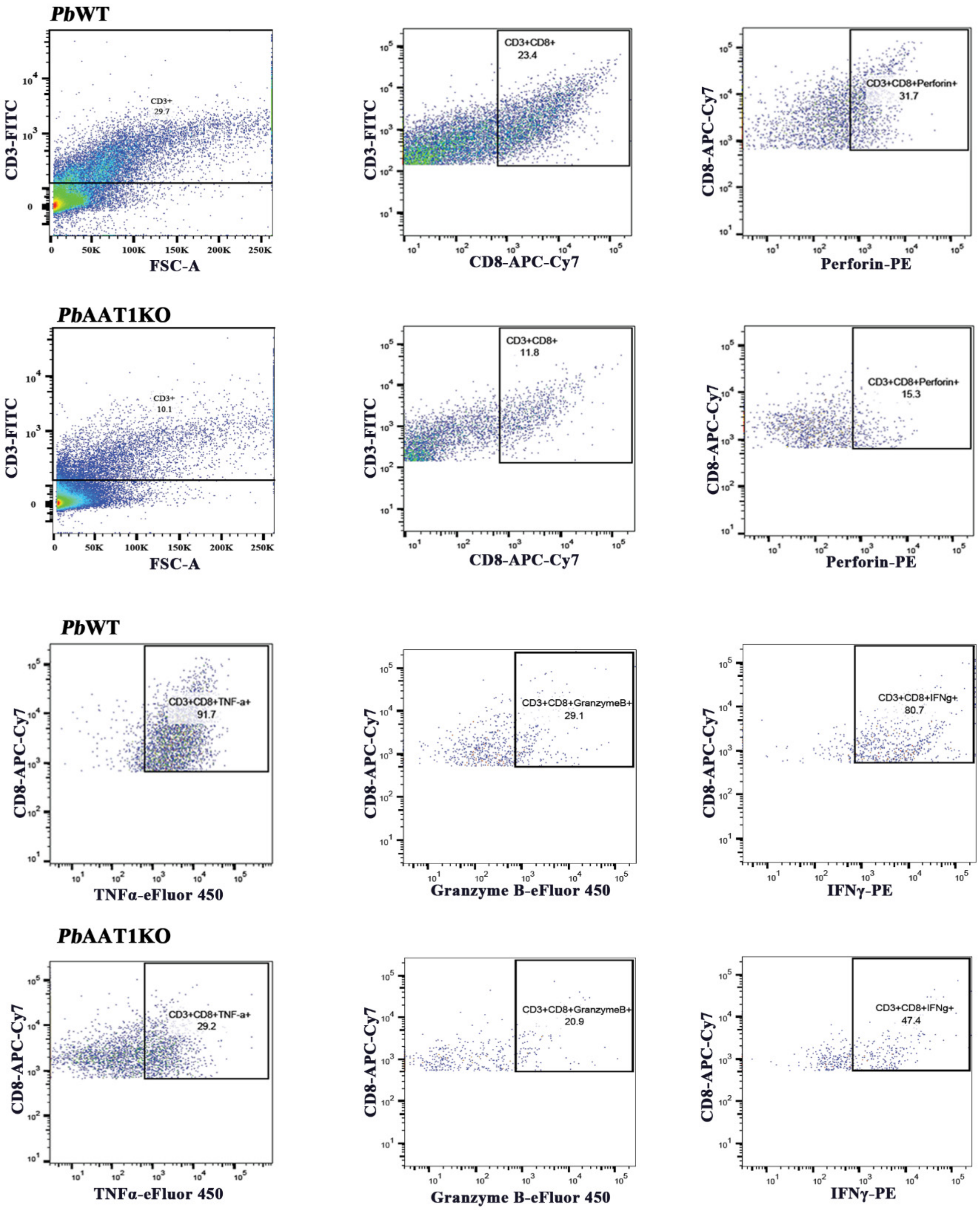

Fig. S5: Flow cytometry analyses of brain isolated T cell population in *Pb*WT- and *Pb*AAT1KO-infected mice. The dot plots provided represent the $CD3^{+}$ T cells, $CD3^{+}CD8^{+}$ T

cells, and $CD3^+CD8^+$ T cells expressing perforin/ TNFα/ granzyme-B/ IFNγ obtained from *Pb*WT- and *Pb*AAT1KO-infected mice brain on day 7 post-infection.

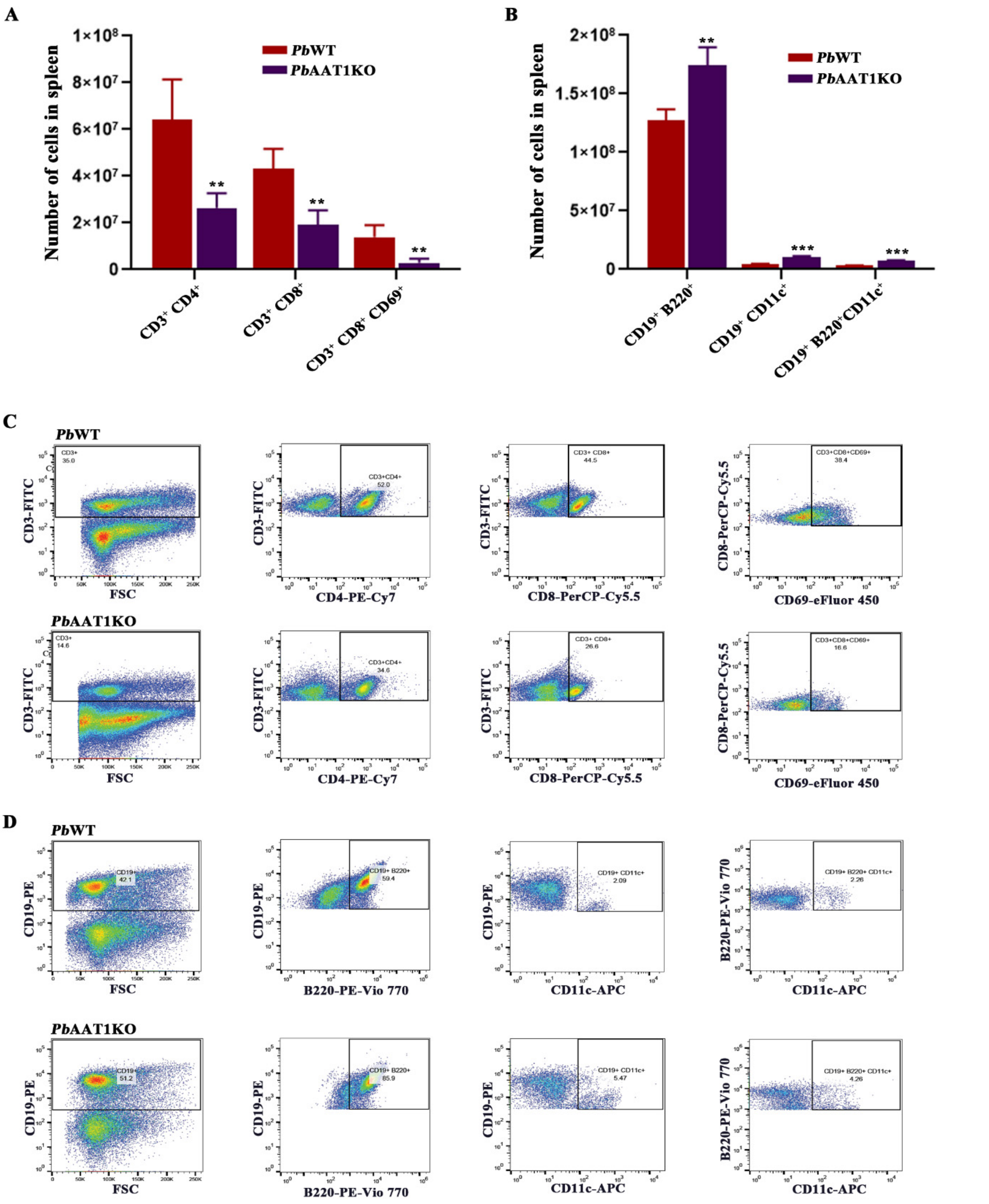

Fig. S6: Flow cytometry analyses of T cell and B cell populations isolated form the spleen of *Pb*WT- and *Pb*AAT1KO-infected mice. (A) Number of splenic $CD3^+CD4^+$ and $CD3^+CD8^+$

double positive T cells, and $CD3^+CD8^+CD69^+$ triple positive T cells obtained from *Pb*WT- and *Pb*AAT1KO-infected mice on day 8 post-infection. The data represent (mean ± SD) two different batches of three mice each (****P*<0.001, unpaired t-test; two-sided). **(B)** Number of splenic $CD19^+B220^+$, $CD19^+CD11c^+$ and $CD19^+B220^+CD11c^+$ B cells obtained from *Pb*WT- and *Pb*AAT1KO- infected mice on day 7 post-infection. The data represent (mean ± SD) two different batches of three mice each (****P*<0.001, unpaired t-test; two-sided). **(C)** Dot plots representing $CD3^+CD4^+$ and $CD3^+CD8^+$ double T positive cells, and $CD3^+CD8^+CD69^+$ triple T positive cells obtained from *Pb*WT- and *Pb*AAT1KO-infected mice spleen. **(D)** Dot plots representing $CD19^+B220^+$, $CD19^+CD11c^+$ and $CD19^+B220^+CD11c^+$ B cells obtained from *Pb*WT- and *Pb*AAT1KO-infected mice spleen.

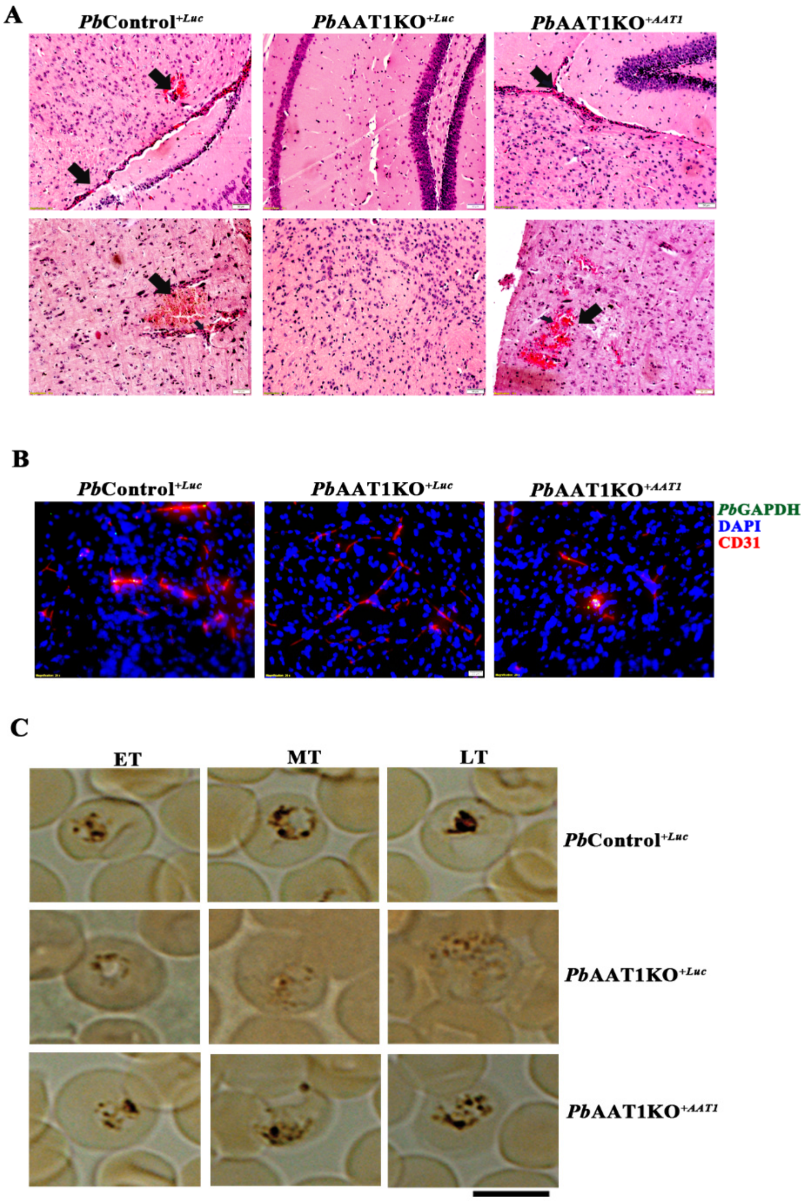

Fig. S7: Cerebral pathogenesis in *Pb*AAT1KO^{+AAT1}-infected mice and bright field images of *Pb*AAT1KO^{+AAT1} parasites. (A) H & E staining of brain sections from *Pb*Control^{+Luc}-, *Pb*AAT1KO$^{+\text{Luc}}$- and *Pb*AAT1KO^{+AAT1}-infected mice. Black arrows represent intracerebral hemorrhages and thrombosed vessels. Images were captured using 10x objective. Scale bar = 50 µm. **(B)** Accumulation of parasitized-RBCs in the CD31^{+} brain vasculature of *PbControl*$^{+Luc}$-, *Pb*AAT1KO$^{+\text{Luc}}$- and *Pb*AAT1KO$^{+\text{AAT1}}$-infected mice. Images were captured using 20x objective.

Scale bar = 50 µm. **(C)** Bright field images of paraformaldehyde-fixed RBCs containing *Pb*Control^{+Luc}*,* *Pb*AAT1KO^{+AAT1} *and* *Pb*AAT1KO^{+Luc} parasites. Images were captured using 100x objective. Scale bar = 5 µm.

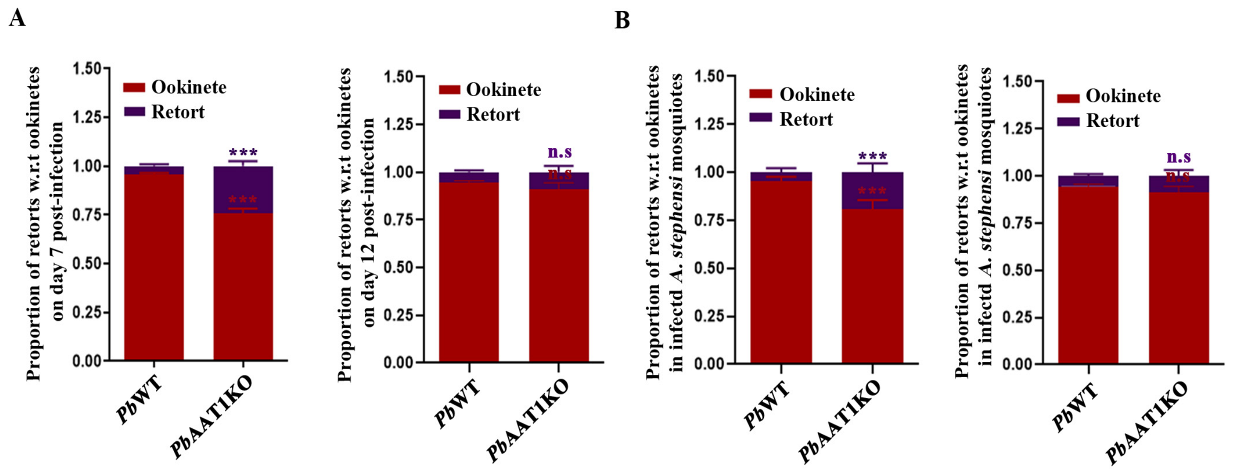

Fig. S8: ***In vitro*** **and** ***in vivo*** **analyses of** ***Pb*****AAT1KO retorts. (A)** Proportion of *Pb*WT and *Pb*AAT1KO retorts with respect to ookinetes formed *in vitro* from day 7 and day 12 post-infection blood collected from *Pb*WT and *Pb*AAT1KO-infected mice. The data represent (mean ± SD) three mice in each group (***P<0.001, n.s - not significant; Two-way ANOVA). **(B)** Proportion of *Pb*WT and *Pb*AAT1KO retorts with respect to ookinetes formed *in vivo* in mosquitoes fed with *Pb*WT- and *Pb*AAT1KO-infected mice on day 7 and day 12 post-infection. The data represent (mean ± SD) ten mosquitoes in each (***P<0.001, n.s - not significant; Two-way ANOVA).

Supplementary Table 1: Details of the primers used for PCR and qPCR analyses.

Primers	5’-3’ Sequence
*Pb*AAT1-F	GCCAGGATCCATGGCTAATAATATAGATATATTAGATTATTGTC
*Pb*AAT1-R	GCCCAAGCTTCTAGTATATTATAATAAATGCGGTAACTATAGATG
GFP-F(pL0027)	ATGGTGAGCAAGGGCGAGGAGCTGTTC
L665	GTTGAAAAATTAAAAAAAAAC
L740	CTAAGGTACGCATATCATGG
*Pb*AAT1-I-F	GCCAGGATCCATGAGTTTATTACCAGAAAAAAACATTTCATTATTTAAAAT
*Pb*GAPDH (F)	ATGGCAATAACAAAAGTCGGAATTAATGG
*Pb*GAPDH (R)	TTAATTTTTGGTGATGTGGATAGCCAAATC
Mouse GAPDH (F)	AAGGTCATCCCAGAGCTGAA
Mouse GAPDH (R)	CTGCTTCACCACCTTCTTGA
Mouse CXCL9 (F)	GCCATGAAGTCCGCTGTTCT
Mouse CXCL9 (R)	GGGTTCCTCGAACTCCACACT
Mouse CXCL10 (F)	GACGGTCCGCTGCAACTG
Mouse CXCL10 (R)	GCTTCCCTATGGCCCTCATT
Mouse CCR7 (F)	TCATTGCCGTGGTGGTAGTCTTCA
Mouse CCR7 (R)	ATGTTGAGCTGCTTGCTGGTTTCG
Mouse CCL2 (F)	GTCACCTGCTGCTACTCATTC
Mouse CCL2 (R)	GCTTGAGGTGGTTGTGGAAAA
Mouse CCL5 (F)	CATCCTCACTGCAGCCGCC
Mouse CCL5 (R)	CCAAGCTGGCTAGGACTAGAG
Mouse CCL19 (F)	ATGTGAATCACTCTGGCCCAGGAA
Mouse CCL19 (R)	AAGCGGCTTTATTGGAAGCTCTGC
Mouse TNFα (F)	AAGCCTGTAGCCCACGTCGTA
Mouse TNFα (R)	GGCACCACTAGTTGGTTGTCTTTG
Mouse IFNγ (F)	TCAAGTGGCATAGATGTGGAAGAA
Mouse IFNγ (R)	TGGCTCTGCAGGATTTTCATG
Mouse Granzyme-B (F)	CCTCCTGCTACTGCTGAC
Mouse Granzyme-B (R)	GTCAGCACAAAGTCCTCTC
Mouse HO-1 (F)	GAGCCTGAATCGAGCAGAAC
Mouse HO-1 (R)	CCTTCAAGGCCTCAGACAAA
*Pb*GAPDH (qPCR F)	AATTAAAGAAGCATCTGAGGGTCCAC
*Pb*GAPDH (qPCR R)	TTGAATATCCCCATTCATTGTCATACC

Supplementary Dataset 1. Peptide summaries of *Pb*WT and *Pb*AAT1KO FVs representing the data provided in Fig. 4D and Fig. S3B and C.
